# Aerosol prime-boost vaccination provides strong protection in outbred rabbits against virulent type A *Francisella tularensis*

**DOI:** 10.1371/journal.pone.0205928

**Published:** 2018-10-22

**Authors:** Katherine J. O’Malley, Jennifer L. Bowling, Elizabeth Stinson, Kelly S. Cole, Barbara J. Mann, Prachi Namjoshi, Karsten R. O. Hazlett, Eileen M. Barry, Douglas S. Reed

**Affiliations:** 1 Center for Vaccine Research, University of Pittsburgh, Pittsburgh, PA, United States of America; 2 Department of Medicine, Division of Infectious Diseases and International Health, University of Virginia, Charlottesville, VA, United States of America; 3 Department for Immunology & Microbial Diseases, Albany Medical College, Albany, NY, United States of America; 4 Center for Vaccine Development, University of Maryland Baltimore, Baltimore, MD, United States of America; University of Rochester, UNITED STATES

## Abstract

Tularemia, also known as rabbit fever, is a severe zoonotic disease in humans caused by the gram-negative bacterium *Francisella tularensis (Ft)*. While there have been a number of attempts to develop a vaccine for *Ft*, few candidates have advanced beyond experiments in inbred mice. We report here that a prime-boost strategy with aerosol delivery of recombinant live attenuated candidate *Ft* S4Δ*aroD* offers significant protection (83% survival) in an outbred animal model, New Zealand White rabbits, against aerosol challenge with 248 cfu (11 LD_50_) of virulent type A *Ft* SCHU S4. Surviving rabbits given two doses of the attenuated strains by aerosol did not exhibit substantial post-challenge fevers, changes in erythrocyte sedimentation rate or in complete blood counts. At a higher challenge dose (3,186 cfu; 139 LD_50_), protection was still good with 66% of S4Δ*aroD*-vaccinated rabbits surviving while 50% of S4Δ*guaBA* vaccinated rabbits also survived challenge. Pre-challenge plasma IgG titers against *Ft* SCHU S4 corresponded with survival time after challenge. Western blot analysis found that plasma antibody shifted from predominantly targeting *Ft* O-antigen after the prime vaccination to other antigens after the boost. These results demonstrate the superior protection conferred by a live attenuated derivative of virulent *F*. *tularensis*, particularly when given in an aerosol prime-boost regimen.

## Introduction

Tularemia (originally known as rabbit fever) is a severe zoonotic infection in humans caused by *Francisella tularensis*, a gram-negative facultative intracellular bacterium [1]. Under current taxonomical classification, there are four subspecies (subsp) of *F*. *tularensis*; *subsp*. *tularensis* (a.k.a. type A) is the most virulent, followed by *subsp*. *holarctica* (a.k.a. type B) and *subsps*. *mediasiactica* and *novicida* which are highly attenuated or avirulent in humans [2] (it should be noted there is some controversy whether *F*. *novicida* is a subsp of *F*. *tularensis*). Tularemia is readily treatable with antibiotics, with an overall case fatality rate less than 2%. Antibiotic resistance, either natural or engineered, is a concern; in the pre-antibiotic era fatality rates reached as high as 30–60% with the pneumonic and septicemic forms of the disease [1]. Once the organism has entered the host, it can disseminate throughout the body and infect a wide array of cell types including macrophages, dendritic cells, epithelial cells, hepatocytes and even erythrocytes. Lung, liver, spleen, and bone marrow are primary targets for bacterial replication and pathogenesis although all organs can be affected, including heart and brain [3–8]. The widespread infection is thought to induce septic shock, disseminated intravascular coagulation and/or acute respiratory distress syndrome resulting in multi-organ failure and death [1, 6].

Although not naturally spread person-to-person by respiratory transmission, *F*. *tularensis* is highly infectious when aerosolized. In the 1950s and 1960s, tularemia was one of the most common laboratory acquired infections, along with brucellosis and tuberculosis [9, 10]. Clinical trials conducted at the time determined that inhalation of a small particle aerosol (≤ 5 µm mass median aerodynamic diameter) containing as few as 15 organisms was sufficient to cause disease in humans[11]. Outbreaks of pneumonic tularemia in landscapers on Martha’s Vineyard have been dubbed “lawnmower tularemia” [12, 13]. Both the former Soviet Union and the United States of America (prior to 1969) evaluated and developed *F*. *tularensis* as a biological weapon [1]. *F*. *tularensis* is considered to be a ‘tier 1’ select agent, pathogens considered to have the highest potential for causing severe morbidity or mortality in humans and animals in the event of an intentional release by terrorists or a rogue nation [14].

A concerted effort was made in the 1950s and 1960s to develop a vaccine for tularemia [15]. Killed, whole cell vaccines proved to be ineffective against lab-acquired tularemia. The Soviet Union developed an attenuated type B strain that was protective as a vaccine. This strain was given to the United States, where investigators passaged it further and dubbed it the “Live Vaccine Strain” (LVS). In clinical trials, LVS given by scarification gave good protection against aerosol challenge with doses ranging from 200–2,000 cfu (10–100 human infectious doses) of a virulent type A strain of Ft, SCHU S4 [11, 16]. LVS-mediated protection was not as good against higher aerosol challenge doses (20,000 cfu) but was still better than killed vaccines. Across all the parenteral LVS vaccine studies, all vaccine recipients seroconverted but not all controls developed disease, so protection is less than the desired 80%. Aerosol LVS vaccination regimens provided superior protection to intradermal inoculation in both monkeys and humans, including at high challenge doses [17–20]. LVS is currently given to at-risk laboratory personnel as an Investigational New Drug but has not been licensed, due to concerns regarding protection and safety/reversion [21]. There are ongoing efforts to re-evaluate LVS as a tularemia vaccine [22, 23] but other approaches are also being considered, including subunit vaccines, vectored vaccines, and new attempts at generating attenuated strains derived from type A.

Among the challenges in developing a tularemia vaccine is defining immune mechanisms and immune correlates that predict protection against aerosol challenge. Because *F*. *tularensis* is a facultative intracellular bacterium, cellular immune responses are thought to be of prime importance in protection. Indeed, experiments in mice have shown that T cell production of IFN-γ or control of bacterial replication in infected macrophages are associated with protection [24, 25]. A recent paper has shown that cell-mediated immune responses can also be seen in human PBMC from LVS recipients bolstering the potential for measuring cellular immune responses in a clinical trial [26]. The vast majority of licensed vaccines, however, measure antibody responses as an immune correlate of protection [27]. Studies in animals and humans have shown a role for antibody in protection against *F*. *tularensis*. Passive immunization with sera from human LVS vaccinees can protect mice against LVS challenge [28]. A study in the rat model demonstrated that passive transfer of antibody from LVS-vaccinated rats reduced the severity and duration of disease caused by intratracheal challenge with SCHU S4 [29]. In most of these antibody studies, the antibody response was directed at the lipopolysaccharide of *F*. *tularensis*, in particular against the O-antigen. While identifying the underlying mechanisms is important for understanding how a particular vaccine protects, translation to humans will be key in demonstrating comparability between results in animal models with those seen in clinical trials that will be essential for licensure under the FDA’s Animal Rule [30].

With the development of systems for genetically manipulating the *F*. *tularensis* genome, attempts have been made to produce a defined attenuated vaccine strain that would not be able to revert to virulence [31–34]. Some of these vaccine candidates have shown success in inbred mouse models; few have been advanced into larger animals. We have previously reported that isogenic mutants (S4Δ*aroD* and S4Δ*guaBA*) of SCHU S4 protected rabbits against respiratory tularemia better than LVS when given as a single vaccination by scarification, although protection was not complete [35]. Based on those prior studies, we here sought to evaluate whether a prime-boost vaccination regimen utilizing S4Δ*aroD* or S4Δ*guaBA* would improve survival of rabbits after aerosol SCHU S4 challenge.

## Results

### Response to prime-boost vaccination regimens

**Fig 1:**
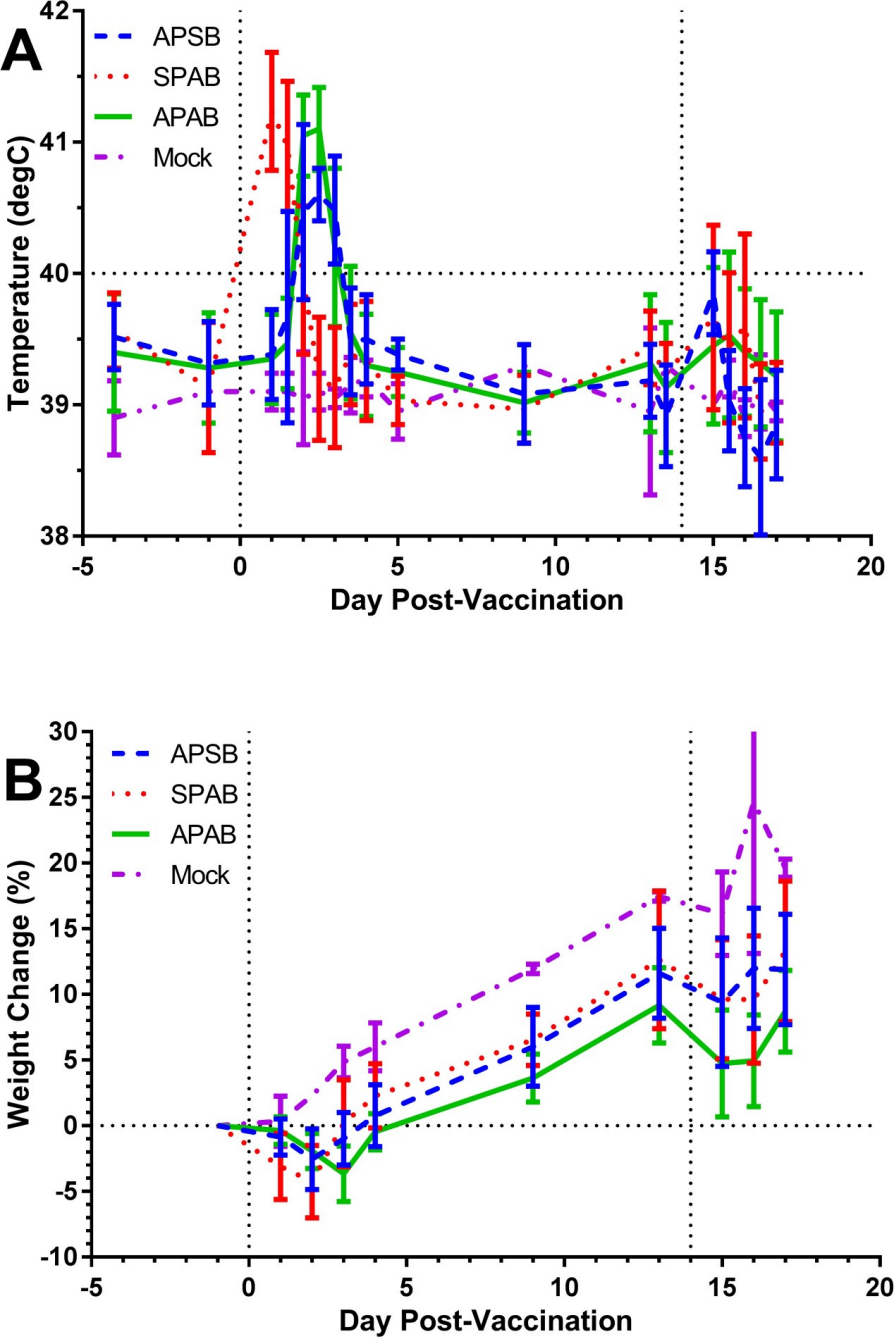
Aerosol prime-boost vaccination of rabbits with S4Δ*aroD* results in transient fever and weight loss. Rabbits were vaccinated with a prime and boost of S4Δ*aroD* by aerosol or scarification. Mock = mock vaccinated controls, APSB = aerosol prime, scarification boost; SPAB = scarification prime, aerosol boost; APAB = aerosol prime, aerosol boost. Graphs show averaged daily group results (n = 6 per vaccine group and 2 mock-vaccinated controls); error bars are not shown for the sake of clarity. Vertical dotted lines indicate days for prime (day 0) and boost (day 14). **A)** Average fever response after prime (day 0) and boost (day 14) with S4Δ*aroD* compared to mock-vaccinated controls. The horizontal dotted line at 40°C indicates the threshold used for fever. By one-way ANOVA, temperature was significantly elevated in the SPAB group on day 1 from all other groups (*p*<0.0001) and on day 3 for the APAB and APSB groups from the SPAB and Mock groups (*p* = 0.0009 for APSB vs SPAB, *p* = 0.0107 for APSB vs Mock; *p* = 0.0073 for APAB vs SPAB, *p* = 0.0448 for APAB vs Mock). Fevers induced by booster vaccination were not significantly elevated over Mock-vaccinated. **B)** Average weight change after vaccination with S4Δ*aroD*. A horizontal dotted line is shown at 0% weight change to make it easier to see where weight was lost in the vaccine groups.

Twenty rabbits were divided into three groups of 6 rabbits with 2 rabbits used as mock-vaccinated rabbits. Mock-vaccinated rabbits were inoculated with Brain Heart Infusion broth. Rabbits were vaccinated with S4Δ*aroD* by scarification or aerosol and then boosted 14 days later with S4Δ*aroD* by the alternate route. One group received both prime and boost vaccinations by aerosol. The groups and doses achieved for each vaccination are shown in Table 1. Fig 1 shows the physiological response to the vaccination. In agreement with our prior results when rabbits were inoculated via scarification with attenuated strains of SCHU S4 [35], the first inoculation of S4Δ*aroD* induced a fever response regardless of route (Fig 1A). Fever (defined as body temperatures Δ 40°C) began within a day post-inoculation of rabbits with S4Δ*aroD* via scarification but was delayed in the aerosol S4Δ*aroD*-vaccinated rabbits. Regardless of route, fever persisted for 1–2 days in vaccinated rabbits and then returned to baseline temperatures. A modest increase in temperature was seen with the booster vaccination on day 14 in all three groups with no discernment between groups in the onset or duration of the response to the boost. Regardless of route of inoculation, S4Δ*aroD*-vaccinated rabbits lost 1–5% of their body weight between 2–4 days after the first vaccine dose (Fig 1B). By day 5 post-prime, all S4Δ*aroD*-vaccinated rabbits were at or above baseline. From that point forward, S4Δ*aroD*-vaccinated rabbits gained weight at the same rate as mock-vaccinated controls. On the day rabbits were boosted, all rabbits including the mock-vaccinated rabbits lost a slight amount of weight again but quickly rebounded.

**Table 1:** Routes and doses for S4ΔaroD prime-boost vaccination groups.

Group	Prime		Boost		Challenge
	Route	Dose*	Route	Dose*	Dose^δ^
APSB^£^	Aerosol	6.7	Scarification	9.6	207 (9)
SPAB^£^	Scarification	8.2	Aerosol	7.9	259 (11)
APAB^£^	Aerosol	6.8	Aerosol	7.9	455 (20)
Mock	Scarification	0	Scarification	0	343 (15)

*median dose, in log_10_ cfu

^£^ APSB = aerosol prime, scarification boost; SPAB = scarification prime, aerosol boost; APAB = aerosol prime, aerosol boost.

^δ^median dose in cfu; (rabbit aerosol *Ft* SCHU S4 LD_50_)

### Survival after aerosol challenge with SCHU S4

**Fig 2:**
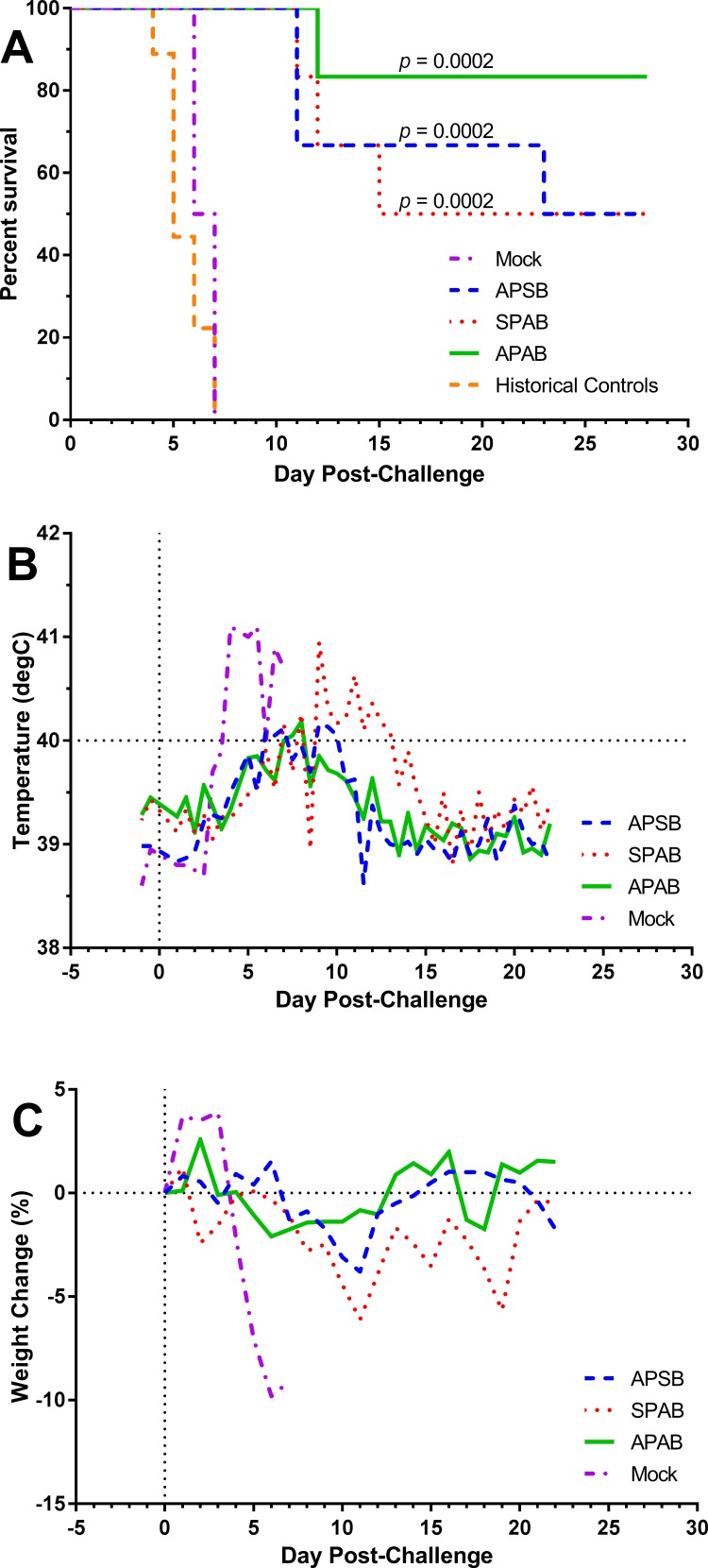
Prime and boost vaccination with S4ΔaroD provides strong protection against aerosol challenge with SCHU S4. Rabbits were vaccinated with a prime and boost of S4Δ*aroD* by aerosol or scarification prior to aerosol challenge with a median dose of 248 cfu SCHU S4 (range: 77–994 cfu). Thirty days post-boost, the rabbits were challenged with SCHU S4. Survival was tracked for 28 days post-challenge; body temperature and weight were tracked until day 22, when surviving rabbits had returned to baseline values. Mock = mock vaccinated controls (n = 2); APSB = aerosol prime, scarification boost (n = 6); SPAB = scarification prime, aerosol boost (n = 6); APAB = aerosol prime, aerosol boost (n = 6). Historical naïve or mock-vaccinated controls given equivalent challenge doses are also shown (n = 9). **A)** Percent survival in each group after aerosol challenge with 248 cfu SCHU S4. Kaplan Meier analysis indicated survival of all three vaccine groups was significantly better than mock-vaccinated rabbits (including historical controls). APAB: 83% survival, *p* = 0.0002; SPAB: 50% survival, *p* = 0.0002; APSB: 50% survival, *p* = 0.0002. **B)** Mean fever response to aerosol challenge with SCHU S4. **C)** Mean weight loss after aerosol challenge with SCHU S4.

Thirty days after the booster vaccination, rabbits were challenged by exposure to a small particle aerosol containing SCHU S4 (median inhaled dose: 248 cfu / 11 LD_50_; range: 77–994 cfu). Mock-vaccinated controls succumbed rapidly to the infection, within 7 days post-exposure all had become moribund and were euthanized (Fig 2A). This is within the range we have seen in the past for naïve or mock-vaccinated rabbits (4–7 days). In all three prime-boost groups, significant (*p* < 0.0002) protection was seen with 83% of rabbits surviving in the APAB group and 50% of rabbits surviving in both the APSB and SPAB groups when compared with mock-vaccinated and historical controls (Fig 2A). In agreement with our previous studies, mock-vaccinated controls developed a severe fever (≥41°C) post-exposure which peaked on days 4 or 5 post-exposure and then dropped before the animals succumbed to the disease (Fig 2B). Similarly, beginning on day 4 substantial weight loss (defined as ≥ 5%) was seen in the mock-vaccinated group that continued to decline until the rabbits succumbed (Fig 2C). The response to challenge in the vaccinated groups, however, was different than what we had seen previously with rabbits vaccinated once by scarification[35]. In previous vaccine studies, surviving rabbits generally developed clinical signs of disease including fever and weight loss in the post-exposure period between days 4–10 before recovering. Here, averaged daily temperatures in the prime-boost groups showed only slight fever responses (≥40°C but ≤41°C). Between days 9–13, fever was seen in the SPAB group where in the APAB and APSB groups fever was only seen on days 7 and 8. Similarly, the average daily weight loss in the prime-boost vaccine groups was less than 5% for only two time points in the SPAB group and not at all for the APAB or APSB groups. Between the three vaccine groups, the febrile response was greatest in the SPAB group and lowest in the APAB group although these differences were not significant (S1 Fig).

**Fig 3:**
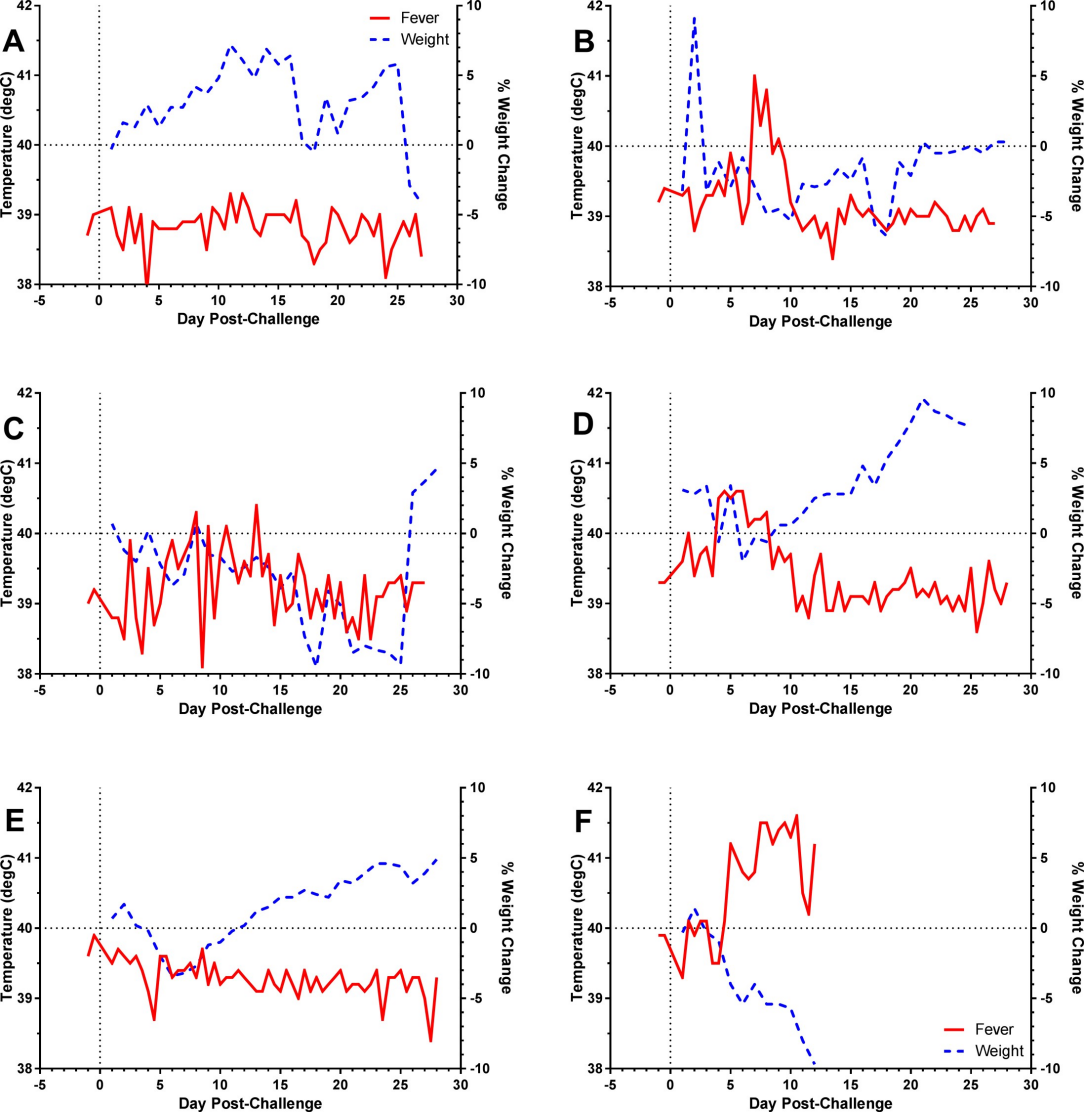
Aerosol challenge with SCHU S4 causes minimal fever or weight loss in individual rabbits vaccinated by APAB with S4ΔaroD. Graphs show daily temperature (solid lines) and weight (dotted lines) for six individual APAB-vaccinated rabbits after aerosol challenge (d0) with SCHU S4. **A-E)** Data from five APAB-vaccinated rabbits which survived challenge; **F)** Data from the one APAB-vaccinated rabbit which succumbed to challenge.

The lack of a noteworthy fever response or weight loss in the vaccine groups, particularly the aerosol primed groups, was surprising. We examined this more closely by breaking down the fever and weight loss changes to individual rabbits in the APAB group, since this group had the highest survival (Fig 3). Within that group, five rabbits survived to 28 days post-challenge while one rabbit succumbed on day 12. Of the five APAB rabbits that survived challenge, two had no fever (Fig 3A and 3E) while one had fever at only 2 points after challenge (Fig 3C). A sustained fever of more than 1 day was seen in only 2 survivors (Fig 3B and 3D). The APAB rabbit that succumbed developed a non-resolving fever on day 5 with fever peaking over 41°C (Fig 3F). Day 5 was approximately the same time that substantial weight loss (>5%) was first recorded. Similar results were seen when looking at weight loss. Only two of the five surviving APAB rabbits had weight loss >5% (from baseline) for more than 1 day post-challenge. The APAB rabbit that succumbed had steady weight loss that began with the onset of fever.

**Fig 4:**
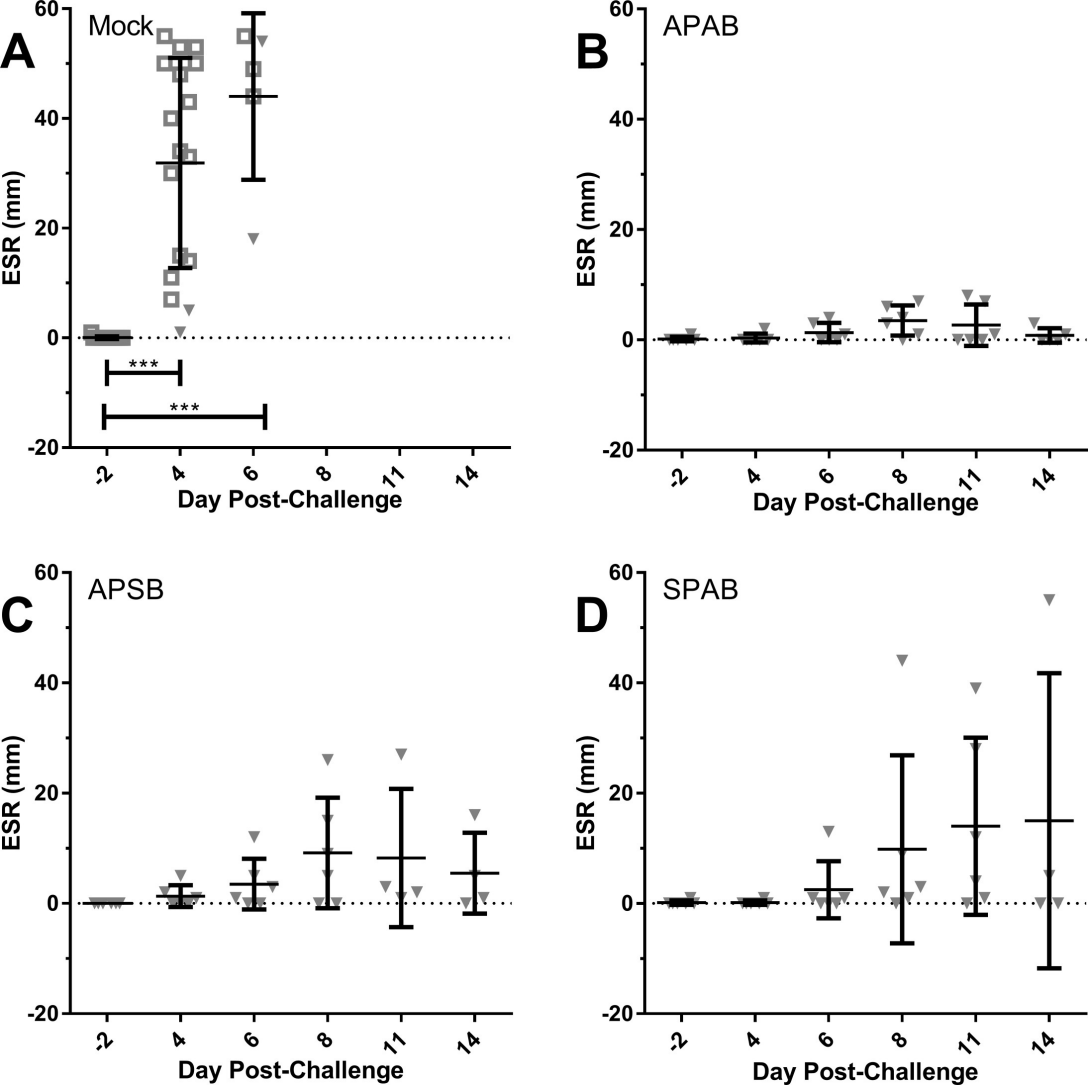
Erythrocyte sedimentation rates (ESR) are lower in vaccinated rabbits post-aerosol challenge with SCHU S4. Graphs show ESR for individual rabbits (gray triangles) with the mean (black center line) and standard deviation (error bars) on different days for rabbits that were **A)** mock-vaccinated, **B)** APAB vaccinated with S4Δ*aroD*, **C)** APSB vaccinated with S4Δ*aroD*, or **D)** SPAB with S4Δ*aroD*. Mock-vaccinated includes historical controls, shown as open squares. Bars with *** indicate where differences were statistically significantly different from baseline as measured by one-way ANOVA, *p* < 0.0001.

At set times pre- and post-challenge, blood was collected from rabbits and samples used to measure bacteremia as well as changes in complete blood count (CBC) and erythrocyte sedimentation rate (ESR) as a measure of the inflammatory response. Bacteremia levels were generally low or non-existent, even in mock-vaccinated rabbits at all time points tested (S2 Fig). In mock-vaccinated rabbits (which includes historical controls) there was a sharp rise in ESR on days 4 and 6 post-infection; these values were significantly higher than baseline (*p* < 0.0001) (Fig 4A). The average ESR for mock-vaccinated rabbits on day 4 was 32 mm, rising to 44 mm on day 6. In the APAB group, ESR did not rise above 10 mm at any time point post-challenge (Fig 4B). ESR levels were mildly elevated in the APSB and SPAB groups after day 4 but the average was less than 20 mm (Fig 4C and 4D). At no point post-challenge were ESR values in vaccinated rabbits significantly higher than baseline values and collectively, it suggested that ESR results predict survival. However, this is not absolute as some rabbits with high ESR survive and others with low ESR succumb. In particular, mock-vaccinated rabbits with low ESR values on day 4 still succumbed to infection.

CBC results showed a decrease in WBC at day 4 in the mock-vaccinated rabbits and in the APAB rabbits (S3 Fig). This drop in WBC was entirely due to a loss of lymphocytes, with no substantive change in granulocytes and macrophages (S4–S6 Figs). In contrast, elevations in granulocytes were seen in rabbits from all three vaccine groups (S5 Fig). Platelet counts remained largely unchanged in vaccinated rabbits while in mock-vaccinated rabbits there was a decrease in platelet counts on day 4 below the level considered indicative of thrombosis in humans (150x10^3^/µl) (S7 Fig). ESR and CBC results are consistent across all vaccine groups and with the lack of a febrile response suggest minimal or no inflammatory response to challenge in vaccinated prime-boost survivors. In keeping with that, gross pathology of lungs and spleens from rabbits found considerable pathology in mock-vaccinated or vaccinated rabbits that succumbed to challenge but no obvious pathology in lungs or spleens from prime-boost vaccinated survivors (S8 Fig).

### Protection against higher lethal aerosol challenge doses

**Fig 5:**
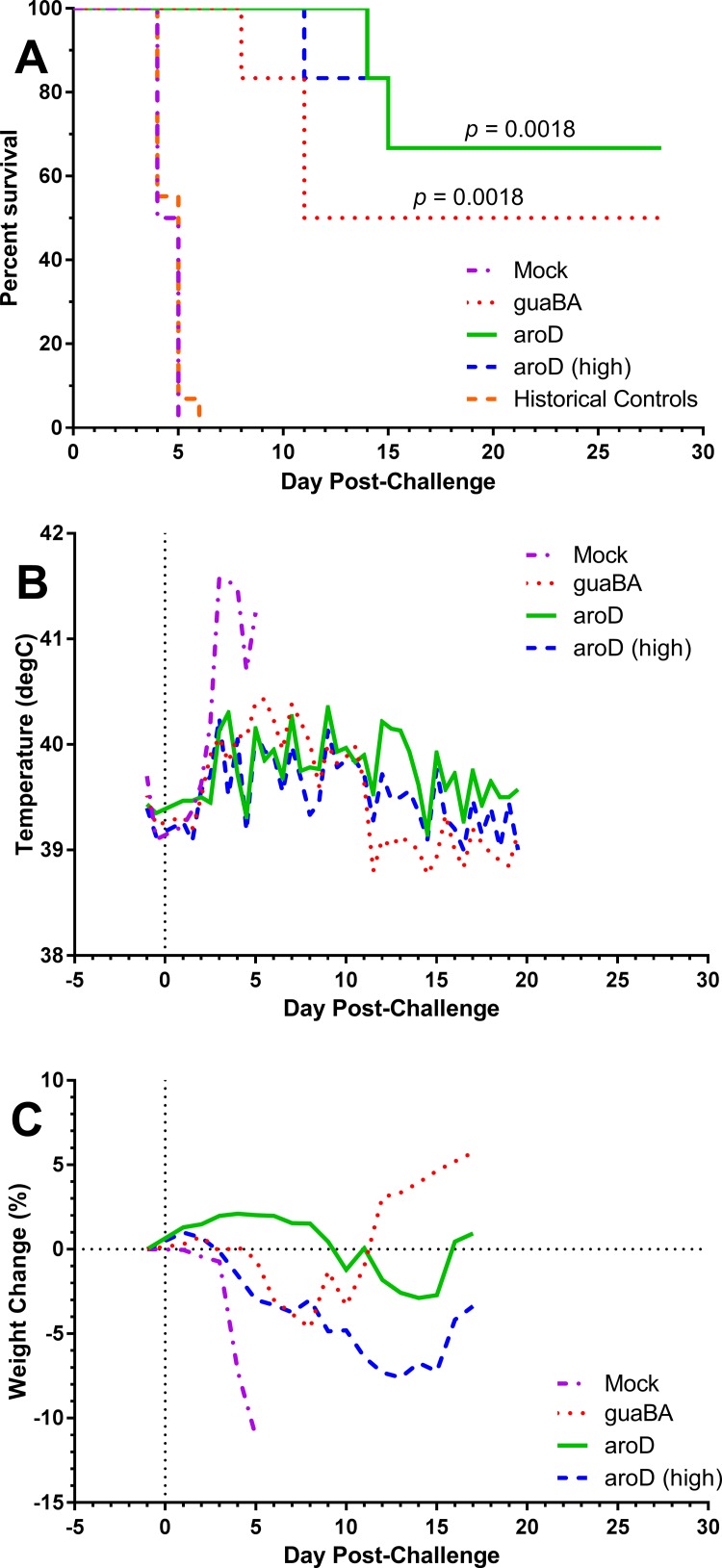
Prime-boost vaccination improves survival at higher SCHU S4 challenge doses. Rabbits were vaccinated by APAB with S4Δ*aroD* or S4Δ*guaBA* prior to aerosol challenge with SCHU S4. Graphs show survival among rabbits vaccinated with S4Δ*aroD* and S4Δ*guaBA* and challenged with 1,475 cfu (solid and dotted lines, respectively) as well as S4Δ*aroD* vaccinated rabbits challenged with 6,316 cfu (designated *aroD* (high), dashed line). Survival was tracked for 28 days post-challenge; body temperature and weight were tracked until day 18–21, when rabbits had returned to baseline values. Mock-vaccinated rabbits are also shown. Graphs show **A)** survival post-challenge. By Kaplan-Meier analysis, survival in all three vaccine groups was significantly different from mock vaccinated controls. S4Δ*guaBA*: 50% survival, *p* = 0.0018; S4Δ*aroD*: 66% survival, *p* = 0.0018; S4Δ*aroD*(high challenge dose): 66% survival, *p* = 0.0018. Historical naïve or mock-vaccinated controls given equivalent challenge doses are also shown (n = 29). **B)** average body temperature by group post-challenge, and **C)** average weight loss by group post-challenge.

We sought to repeat these promising results for APAB S4Δ*aroD* vaccination against a higher challenge dose as well as evaluate whether a similar improvement in protection could be seen with S4Δ*guaBA* using an APAB regimen, another vaccine that had shown promising results when given once by scarification. In addition, because the initial prime-boost vaccination study used a lower challenge dose (~10 LD_50_) we included a third APAB vaccine group given S4Δ*aroD* to evaluate protection against an even higher challenge dose (~300 LD_50_). Eighteen rabbits were split into three groups of six; one group received S4Δ*guaBA* while the other two groups received S4Δ*aroD*. Vaccination doses are shown in Table 2. For each challenge dose we included 2 control, mock-vaccinated rabbits. As we had seen before, vaccination was well tolerated by the rabbits with minimal, transient fever and weight loss after delivery of either the prime or boost (S9 Fig). Thirty days after the second vaccination, the S4Δ*guaBA* & S4Δ*aroD* rabbits (and associated controls) were aerosol challenged with SCHU S4 at a median dose of 1,475 cfu (64 LD_50_) and the higher challenge dose S4Δ*aroD* group and associated controls received 6,316 cfu (275 LD_50_). As expected from prior studies, mock-vaccinated rabbits at both challenge doses succumbed within 4–5 days of infection. In the ‘low’ challenge dose groups, 66% that received S4Δ*aroD* survived challenge while 50% that received S4Δ*guaBA* survived challenge. At the higher challenge dose, 66% that received S4Δ*aroD* survived infection. Similar to what we had observed with the first prime-boost study, rabbits in the vaccine groups at either challenge dose had less fever and weight loss post-challenge compared to the mock-vaccinated rabbits (Fig 5B and 5C).

**Table 2:** Vaccination doses to compare efficacy of S4ΔguaBA and S4ΔaroD as well as efficacy of S4ΔaroD against a high challenge dose.

Vaccine	Prime*	Boost*	Challenge*
S4Δ*guaBA*	7.2	8.7	3.1
S4Δ*aroD*	7.2	8.5	3.1
Mock	0	0	3.4
S4Δ*aroD* (High)^£^	7.6	8.3	3.8
Mock (High) ^£^	0	0	4.0

^£^high challenge dose

*median dose in log_10_ cfu

### Vaccination induces strong plasma IgG responses to SCHU S4

**Fig 6:**
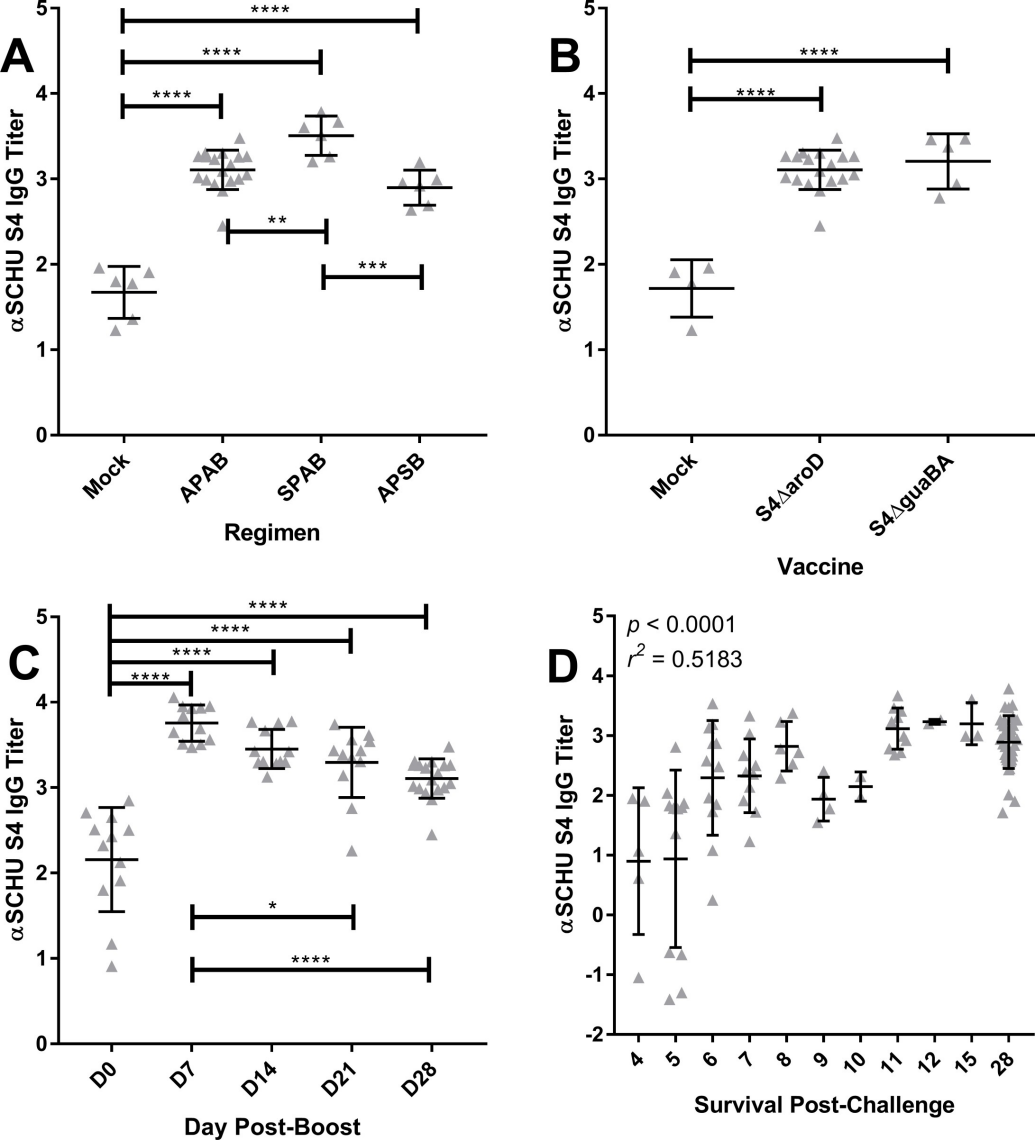
Pre-challenge plasma IgG response to SCHU S4 in vaccinated rabbits corresponds with survival after challenge. Rabbits were bled post-vaccination and the plasma antibody response was assessed by ELISA. Values shown are the median effective concentration (EC_50_) values for plasma IgG responses against heat-killed SCHU S4 in rabbits. Statistical tests were done by one-way ANOVA. **A)** day 28 post-vaccination plasma IgG responses to rabbits vaccinated with S4Δ*aroD* by aerosol prime, aerosol boost (APAB), scarification prime, aerosol boost (SPAB) or aerosol prime, scarification boost (APSB)(n = 6/group). Results from mock-vaccinated rabbits. ** indicates a statistically significant difference by ANOVA, *p* = 0.0067; *** indicates a significant difference, *p* = 0.0006; **** = highly significant differences, p<0.0001. **B)** Graph shows comparison between plasma IgG titers on day 28 post-vaccination for rabbits vaccinated by APAB with S4Δ*aroD* (n = 12) or S4guaBA (n = 6). Mock-vaccinated rabbits are shown for comparison; **** = highly significant differences, *p* <0.0001. **C)** change in plasma IgG titers over time for S4Δ*aroD*; days shown are post-vaccination; * = significant difference, p = 0.0215; **** = highly significant differences, *p* <0.0001.One-way ANOVA analysis was used to determine significant differences between pre- and post-vaccination bleeds; * = significant, p <0.05, **** = highly significant, p<0.0001. **D)** Graph shows day 28 post-vaccination (day -2 relative to challenge) IgG titer (y-axis) compared to survival time (in days) after aerosol challenge with SCHU S4 (x-axis) across all vaccine groups from all studies conducted to date including mock-vaccinated rabbits (n = 107 rabbits, including studies reported here and [35, 36]). ANOVA analysis indicated a linear trend in which higher plasma IgG responses to SCHU S4 predicted longer survival time in days (*p* = 0.0001, *r*^*2*^ = 0.5183).

Having established that live attenuated S4-based vaccines could provide safe, effective protection against S4 in the rabbit model, we sought to determine whether the immune response could predict protection. We previously demonstrated that antibody responses against heat-killed SCHU S4 might be useful for predicting protection [36]. Regardless of the prime-boost regimen used, plasma IgG titers 28 days after vaccination (2 days prior to challenge) with S4Δ*aroD* were significantly higher than mock-vaccinated rabbits (*p* < 0.0001)(Fig 6A). Rabbits given a scarification prime and aerosol boost (SPAB) had higher plasma IgG titers than rabbits that received an aerosol prime, scarification boost (APSB) or both doses by aerosol (APAB), and these differences were statistically significant (*p* = 0.0006 and *p* = 0.0067 for APSB and APAB, respectively). Antibody titers to prime-boost vaccination (regardless of route) were also significantly higher than antibody responses to a scarification prime only (S10 Fig). We next compared plasma IgG titers between rabbits that received an APAB with either S4Δ*aroD* or S4Δ*guaBA* (Fig 6B). Plasma IgG titers in both groups were significantly higher than mock-vaccinated rabbits (*p* < 0.0001) but were not significantly different from one another (*p* = 0.7413). We also looked at earlier time points for plasma IgG titers induced by APAB S4Δ*aroD*. At all timepoints post-vaccination, plasma IgG titers were highly significantly different from pre-vaccination IgG titers (*p* < 0.0001)(Fig 6C). Post-vaccination, plasma IgG titers peaked at day 7 post-vaccination and gradually declined out to day 28. Differences between plasma IgG titers on day 7 post-vaccination were significantly different from day 21 and 28 post-vaccination (*p* = 0.0216 and *p* < 0.0001 for d21 and d28, respectively). We then compared whether plasma IgG titers taken 2 days pre-challenge across all vaccine studies (including re-analyzed plasma from prior studies with a single vaccination only [35, 36]) could predict survival from challenge. Plasma IgG responses to SCHU S4 did correspond with longer survival after challenge; one-way ANOVA was able to determine a significant linear trend (*p* = <0.0001, *r*^*2*^ = 0.5183)(Fig 6D). It should be noted, however, that beyond day 6 survival the trend does flatten somewhat. Plasma IgA responses did not correspond with survival (S11 Fig).

**Fig 7:**
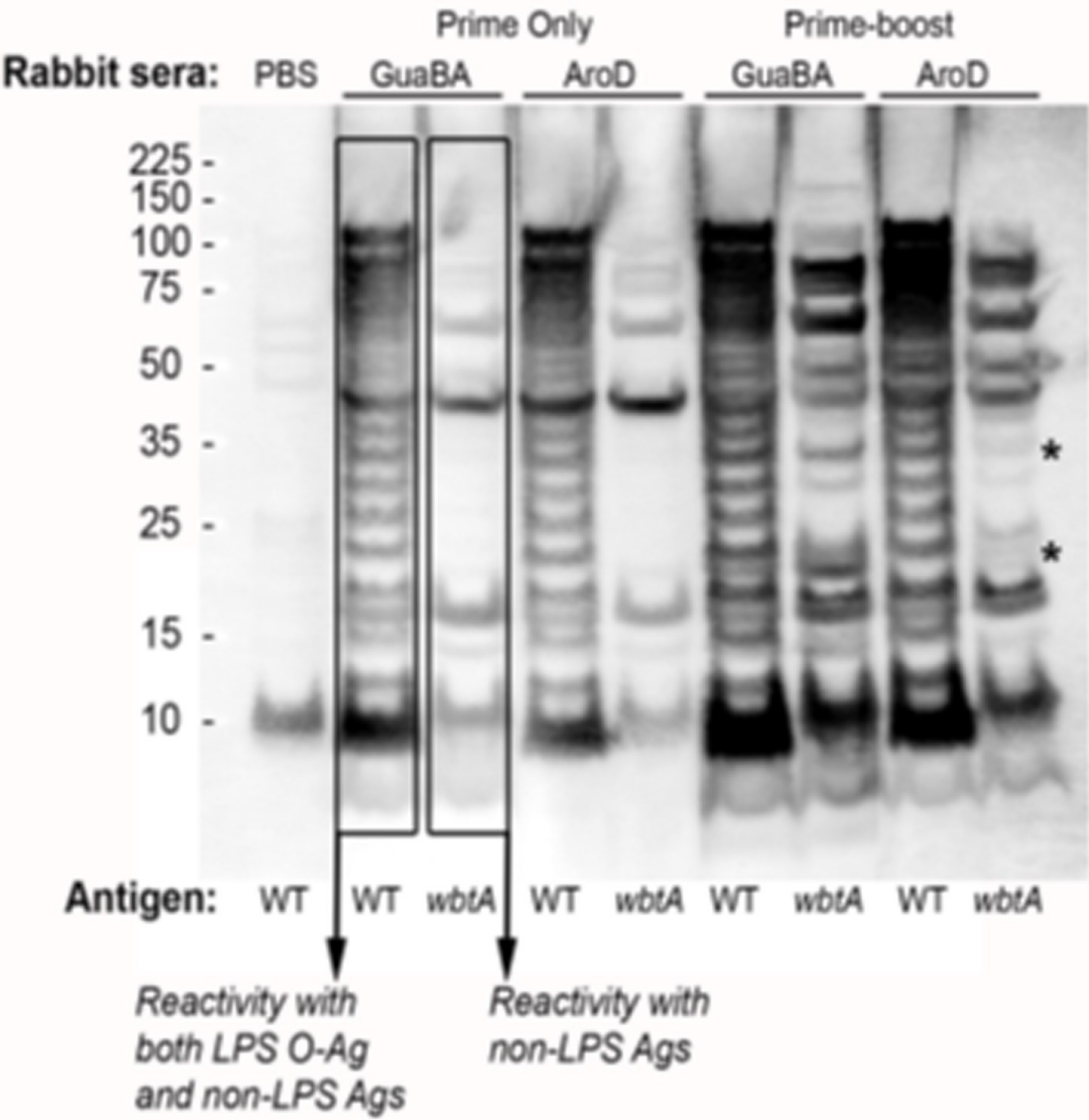
Differences in pre-challenge plasma antibody reactivity to Ft antigens between survivors and non-survivors of S4 challenge. Whole cell lysates of Ft LVS wildtype (WT) and the O-Antigen deficient LVS mutant (*wbtA*) grown in BHI broth were loaded into 9 wells of an SDS-PAGE gel prior to resolution and transfer to a nitrocellulose membrane. The membrane was reversibly stained with amido black and cut into 5 sections with 2 lanes/section. The sections were probed in parallel with the indicated 5 rabbit sera pools prior to simultaneous chemiluminescent development. Western blot immuno-reactivity with the bacterium’s LPS (which is decorated to varying degrees with O-Ag) resolved by SDS-PAGE typically reveals a characteristic LPS ladder pattern in WT Ft (left lanes for each vaccine) but not in Ft deficient for O-Ag such as wbtA (right lanes for each vaccine)[56–58]. * notes bands at ~35 and ~22 kDal that are different between the antibody response to S4Δ*aroD* and S4Δ*guaBA*.

Western blot analysis using lysates of wild type (WT) and O-antigen-deficient (*wbtA*) *Ft* LVS revealed that the plasma IgG response to a single vaccination (prime-only) with either S4Δ*aroD* or S4Δ*guaBA* is largely directed at the bacterium’s O-antigen and a small subset of proteins most readily observed in an O-Ag Ft mutant, *wbtA* (Fig 7). Note that detection of the bacterium’s O-Ag (the repeating carbohydrate moiety found in variable numbers on the bacterium’s heterogeneous LPS) yields a typical “ladder pattern” visible in WT, but not *wbtA*, an O-Ag-deficient, *Ft*. Following a boost, the antibody repertoire broadens to include a greater number of O-Ag-independent antigens (presumed to be proteins) with some modest divergence between the S4Δ*aroD*- and S4Δ*guaBA*-induced Ab responses (compare reactivity of the prime-boost S4Δ*guaBA* and S4Δ*aroD* plasma with the *wbtA* strain in the ~35 and ~22 kDa range). Identification of these putative, differentially-recognized bands and a more-accurate quantitative and qualitative analysis of these Ab responses is ongoing. The results of our antibody ELISA and western blot analyses suggest that antibody responses to specific Ft antigens may be of value as a potential correlate of protection. We are continuing to investigate this question.

## Discussion

We have previously shown that a single vaccination by scarification with deletion mutants of SCHU S4 outperformed LVS in the outbred rabbit model of lethal aerosol SCHU S4 challenge[35]. In those initial studies, LVS only extended time to death while some rabbits vaccinated with the recombinant mutants (S4Δ*aroD* and S4Δ*guaBA*) survived aerosol challenge with SCHU S4 (27 & 36%, respectively). We subsequently demonstrated that the protection elicited by LVS vaccination could be improved by delivering the vaccine by either oral gavage or small-particle aerosols [36]. As the next step, we decided to evaluate whether a prime-boost regimen would further improve vaccine-mediated protection in rabbits. We present data here demonstrating that prime-boost regimens of a deletion mutant of SCHU S4 delivered by aerosol and/or scarification provides significant protection in outbred NZW rabbits against aerosol challenge with SCHU S4. This included a reduction in morbidity as well as survival from challenge. The data further suggests that the combination of aerosol prime and aerosol boost provided the best protection, although these differences were not significant in the experiments reported here. We further demonstrated that the pre-challenge plasma IgG response in vaccinated rabbits corresponded with survival, suggesting antibody could be a useful correlate of protection. Given the level of attenuation and the high level of protection seen against robust challenge doses, S4Δ*aroD* warrants further evaluation as a promising tularemia vaccine candidate.

There is a question whether challenge dose is determining the outcomes we see. Certainly, in the initial experiment reported here we saw higher overall survival with the lower challenge dose (median: 248 cfu; 11 LD_50_) compared to prior studies. However, mock-vaccinated rabbits did not survive challenge and the time to death for mock-vaccinated rabbits was not significantly different from prior studies with higher challenge doses (S1 Table). In the second vaccine study we used higher challenge doses in order to determine whether the protection afforded by prime-boost vaccination would be similar against a more rigorous challenge. There is some variation between individual animals in terms of the vaccination doses and challenge doses; this variation is the result of differences in respiratory function between rabbits that causes them to inhale different volumes of air during aerosols, which were a fixed 10-minute duration. However, there are no significant differences in doses between groups within individual experiments and we have found no correlation between challenge dose and survival of vaccinated rabbits whether comparing individual rabbits or groups of rabbits (by one way ANOVA, *p* = 0.3818, *r*^*2*^ = 0.1195, S1 Table). In fact, in the initial experiment reported here the APAB group had the highest overall median challenge dose (455 cfu) yet had the best overall survival in that experiment.

While it was expected that a prime-boost vaccination regimen would improve protection, we did not expect that delivering both doses by aerosol would be better than the combinations of scarification and aerosol (although this difference did not rise to the level of significance). We thought that scarification would provide stronger systemic immunity while aerosol delivery would provide stronger immune responses at the site of entry. Aerosol-primed rabbits, regardless of the route of boost, had less morbidity than the scarification primed-group. Rabbits primed with *S4*Δ*aroD* by scarification did have higher plasma α-hkSCHU S4 IgG titers on day 28 than rabbits that were primed by aerosol, although this did not improve survival. Based on that and our prior ELISA results against LVS endotoxin, it was surprising then that across all the vaccine studies we have done in rabbits, pre-challenge plasma α-hkSCHU S4 IgG titer on day 28 post-vaccination did roughly correspond with protection post-challenge. Rabbits with low plasma IgG titers (<2.5 log_10_ EC_50_) to whole, heat-killed SCHU S4 did not survive long. Survival time was extended at higher titers, although titers >3log_10_ EC_50_ did not insure survival. IgA titers in plasma did not correspond with survival at all, but it is possible that we would have seen different results had we looked at IgA in mucosal secretions. Data from the literature support a role for both cellular or antibody immune responses in vaccine-mediated protection against *F*. *tularensis*. In some studies, antibody titers have been shown to predict protection and passive immunization has provided at least limited protection in animal studies [28, 37–44]. Other studies have presented contrary findings [11, 44, 45]. Considering that *F*. *tularensis* is a facultative intracellular bacterium yet can have a significant extracellular phase, it seems likely that antibody and cellular immune responses would have complementary roles in a protective response. The results we present here suggest plasma IgG titer may serve as a correlate of protection against tularemia. That does not mean that antibody is the mechanism by which S4Δ*aroD* (or S4Δ*guaBA*) immunized rabbits are protected against SCHU S4.

A more refined approach, looking at specific antigens rather than a crude, killed preparation of bacteria should provide a better answer on the role of antibody. Western blots of plasma IgG response from S4Δ*aroD* and S4Δ*guaBA*-vaccinated rabbits suggested that the prime vaccination generated antibody mostly against O-antigen while the boost vaccination resulted in stronger antibody responses against protein antigens. The identity of these protein antigens and their relationship with rabbit survival is an active area of investigation. Analyses of the immunoproteome to *F*. *tularensis* have been reported by others in mice and humans [39], however to our knowledge this is the first report in a large outbred animal with a challenge component where pre-challenge antibody responses from vaccinated survivors and non-survivors are compared. Considering the overall strength of the antibody response to vaccination in the rabbits, a refined ELISA focusing on specific *F*. *tularensis* antigens, not whole, heat-killed bacteria, may prove to be a better correlate. This is an area we are actively exploring, as well as evaluating antibody in mucosal tissues, antibody subtypes, and functional antibody responses.

Another striking finding was the overall low or non-existent fever and ESR response after challenge in the surviving prime-boost vaccinated rabbits. In our prior study with single vaccinations, all rabbits challenged with SCHU S4 including survivors developed a fever by no later than day 4 post-challenge [35]. In prior studies with naive rabbits, we had seen that lymphopenia and thrombocytopenia, coupled with a rise in ESR, are associated with fatal disease and that this is similar to what has been reported in humans [4]. We saw similar results in the mock-vaccinated rabbits in these studies but not in the prime-boost vaccinated rabbits, a surprising contrast from the prior single vaccination studies. This suggests that the aerosol prime-boost regimen generated very strong, protective immunity with little or no morbidity in a susceptible outbred animal model.

In summary, we have demonstrated here that priming and boosting by aerosol delivery of an attenuated recombinant derivate of SCHU S4 offers strong protection against a virulent Ft aerosol challenge. The level of protection was nearly complete, even at high challenge doses. We would envision pursuing a similar strategy in humans, similar to respiratory delivery of vaccines for influenza or measles [46–49]. Aerosol delivery would build on the time-honored premise of the oral Sabin vaccine, that vaccination at the site of pathogen entry is superior to parenteral inoculation in the protection conferred. Future efforts will build on the results detailed here, particularly to determine the immunological mechanisms and antigens important for this protection.

## Materials & methods

### Biosafety and regulatory information

All work with live *F*. *tularensis* was conducted at biosafety level (BSL)-3 in the University of Pittsburgh Regional Biocontainment Laboratory (RBL). For respiratory protection, all personnel wore powered air purifying respirators (3M GVP-1 PAPR with L-series bumpcap) or used a class III biological safety cabinet. Vesphene II SE (1:128 dilution, Steris Corporation, Erie, PA) was used to disinfect all liquid wastes and surfaces associated with the agent. All solid wastes, used caging, and animal wastes, were steam-sterilized. Animal carcasses were digested via alkaline hydrolysis (Peerless Waste Solutions, Holland, MI). The University of Pittsburgh Regional Biocontainment Laboratory is a Registered Entity with the CDC/USDA for work with *F*. *tularensis*. The University of Pittsburgh’s Biohazard Committee approved these studies. Work with recombinant *Francisella tularensis* strains was approved by the University of Pittsburgh’s Institutional Biosafety Committee.

### Animal studies

All animal work performed for this publication adhered to the Guide for the Care and Use of Laboratory Animals of the National Institutes of Health (NIH) and the Animal Welfare Act (AWA). All studies were performed under protocols approved by the University of Pittsburgh’s Institutional Animal Care and Use Committee (Protocols: 1105929 & 14053602). The University of Pittsburgh is fully accredited by the Association for Assessment and Accreditation of Laboratory Animal Care (AAALAC). Rabbits were monitored at least once daily prior to infection and at least twice daily after infection. For blood collection, rabbits were anesthetized with 2–5% isoflurane and bled from the saphenous vein. Rabbits that were determined to be moribund (any of the following clinical signs: weight loss ≥ 20%, body temperature < 34°C, unresponsive to prodding, respiratory distress) were first anesthetized with isoflurane (2–5%) and then euthanized promptly by barbiturate overdose (100 mg/kg sodium pentobarbital given i.v. or i.c.). Five of the forty-two rabbits (12%) in these studies did succumb prior to administration of euthanasia; this was within the 20% anticipated in the approved protocols.

### Rabbits

Young female New Zealand White (NZW) rabbits (Robinson Services, Inc.) were housed in the University of Pittsburgh Regional Biocontainment Laboratory (RBL) at ABSL-3 for the duration of the studies. Prior to vaccination, IPTT-300 temperature/ID chips (BioMedic Data Systems, Seaford, DE) were implanted subcutaneously. Body weight was recorded once in the morning and body temperature was recorded twice daily. Temperature was read using a DAS-7000 reader (BioMedic Data Systems).

### Bacteria

*F*. *tularensis* SCHU S4 was originally obtained from the Dynport Vaccine Company respectively and were stored as single-passage stocks [4]. *F*. *tularensis* SCHU S4 Δ*aroD* and SCHU S4 Δ*guaBA*, generated as previously described in the laboratories of Dr. Eileen Barry and Dr. Barbara Mann, were shipped to the University of Pittsburgh for these experiments[31, 50–52]. *F*. *tularensis* was grown first on Cysteine Heart Agar (CHA) for two days prior to growing overnight in Brain Heart Infusion (BHI) broth supplemented with ferric pyrophosphate and L-cysteine[53, 54] using baffled, vented polycarbonate Erlenmeyer flasks [4]. Attenuated strains were grown in 200 ml cultures using 1 L flasks, concentrated by centrifuging at 4,000x g for 10 minutes and resuspended in fresh BHI broth for aerosolization.

### Vaccination by scarification

Rabbits were vaccinated by scarification or by aerosol exposure. For scarification, rabbits were anesthetized by subcutaneous injection of ketamine (80 mg/kg) and xylazine (8 mg/kg); once anesthesia was confirmed a small area of the dorsal surface was shaved. Approximately 0.1 ml of bacteria at a concentration of 1x10^10^ cfu/ml were placed in a drop on the shaved area and a bifurcated needle (Becton Dickinson) was jabbed through the drop of bacteria into the skin 17 times. The drop was allowed to absorb into the skin, after which the xylazine was reversed by i.m. injection of 0.2–1 mg/kg yohimibine. The scarification site was monitored daily for the first seven days after vaccination.

### Aerosol exposures (vaccinations and challenge)

Aerosols of *F*. *tularensis* were generated inside a class III biological safety cabinet (Baker Co., Sanford, ME) located inside the RBL as previously described [4]. Briefly, rabbits were exposed two at a time for 10 minutes in a nose-only exposure chamber (CH Technologies, Westwood, NJ) using a 3-jet Collison nebulizer while plethysmography data was collected using Buxco XA or Finepointe software (Buxco Research Systems, Wilmington, NC) during the exposure. After the exposures were completed, nebulizer and all-glass impinger (AGI) contents were quantified on CHA. Aerosol concentration and inhaled (presented) dose were determined as described previously [55]. Mock-vaccinated rabbits were exposed to aerosols of Brain Heart Infusion broth.

### ELISA

ELISA were performed using standard ELISA procedures. Briefly, dilutions of rabbit sera were incubated for 1 hour at 37°C on 96-well plates coated with heat-killed *F*. *tularensis* SCHU S4. Duplicate wells were used for each dilution. A positive control was included on every plate using plasma drawn on day 28 from a vaccinated rabbit that survived SCHU S4 challenge. After washing with PBS-Tween, secondary goat anti-rabbit IgG-HRP (Fitzgerald Industries, Acton, MA) was added to the plates and incubated for 1 hour at 37°C, after which the plates were washed again with PBS-Tween and BM Chemiluminescence ELISA Substrate (Roche Applied Sciences, Indianapolis, IN) was added to the plates. Plates were then read on an Lmax plate reader (Molecular Devices, Sunnyvale, CA). Median effective concentration (EC50) was determined by four-parameter logistical regression of ELISA data using GraphPad Prism 6.

### Erythrocyte sedimentation rates (ESR)

rabbit whole blood collected in EDTA was pipetted using a glass Pasteur pipet into glass Wintrobe tubes. After one hour, the degree of sedimentation was recorded in mm for each rabbit.

### Hematology

rabbit whole blood collected in EDTA was analyzed on a VetScan HM2 (Abaxis, Union City, CA) to determine the white blood cell counts including granulocytes, macrophages, and lymphocytes as well as platelets.

### SDS-PAGE and western blot analysis

Whole cell samples containing 10 μg of *Ft* protein (~1x10^8^ cells) were mixed with Laemmli sample buffer and boiled for 10 min prior to resolution through 4–12% gradient SDS-PAGE pre-cast gels (NuPAGE Bis-Tris, Invitrogen). The running buffer was NuPAGE MES SDS buffer from Invitrogen; gels were run at 120 V. Resolved gels were stained with either Coomassie blue (BioRad) or transferred to nitrocellulose membranes. Coomassie-stained gels were scanned into Adobe Photoshop using an HP LaserJet Pro 300 color (MFP-m375nw) scanner. Membranes were cut into sections, each containing one lane of WT and one lane of *wbtA*. Membrane sections were blocked in parallel for 30 min with PBS, 0.05% Tween 20, 5% non-fat dry milk. Rabbit sera (α-PBS, α-*S4*Δ*guaBA* prime, α- *S4*Δ*aroD* prime, α- *S4*Δ*guaBA* prime-boost, α- *S4*Δ*aroD* prime-boost) were applied in parallel for overnight incubation at a dilution of 1:1000 in PBS, 0.05% Tween 20. HRP-conjugated goat anti-rabbit secondary Ab (Southern Biotech 4010–05) was used at a dilution of 1:5000 in PBS, 0.05% Tween 20. Development of the chemiluminescent substrate (SuperSignal West Pico, Pierce, Rockford, IL) from the 5 individual membrane sections was visualized simultaneously using an BioRad ChemiDoc Touch Imaging System.

### Statistical methods

Data was collected and organized using spreadsheets in Microsoft Excel 2016; graphing and statistical analyses were conducted using GraphPad Prism 7. Historical controls [4, 35, 36] were used for statistical analyses.

## Supporting information

S1 Fig Febrile response to challenge. Graph shows the % of time points post-challenge that were scored as febrile (body temperature ≥ 40oC) for individual rabbits in the different vaccine groups. Individual rabbits that survived challenge are marked with gray triangles while rabbits that succumbed are marked with a gray X. Means and standard deviations are indicated by black bars for each group.
(TIF)

S2 Fig Bacteremia after challenge. Graphs show bacteremia levels (in cfu/ml of blood) for individual rabbits (gray triangles) with the mean (black center line) and standard deviation (error bars) on different days for rabbits that were A) mock-vaccinated, B) APAB vaccinated with S4ΔaroD, C) APSB vaccinated with S4ΔaroD, D) SPAB vaccinated with S4ΔaroD.
(TIF)

S3 Fig Changes in White Blood Cell (WBC) count after challenge in different vaccine groups. Graphs show WBC for individual rabbits (gray triangles) with the mean (black center line) and standard deviation (error bars) on different days for rabbits that were A) mock-vaccinated, B) APAB vaccinated with S4ΔaroD, C) APSB vaccinated with S4ΔaroD, D) SPAB vaccinated with S4ΔaroD. Mock-vaccinated includes historical controls, shown as open squares.
(TIF)

S4 Fig Changes in lymphocyte (LYM) count after challenge in different vaccine groups. Graphs show LYM for individual rabbits (gray triangles) with the mean (black center line) and standard deviation (error bars) on different days for rabbits that were A) mock-vaccinated, B) APAB vaccinated with S4ΔaroD, C) APSB vaccinated with S4ΔaroD, D) SPAB vaccinated with S4ΔaroD. Mock-vaccinated includes historical controls, shown as open squares.
(TIF)

S5 Fig Changes in granulocyte (GRA) count after challenge in different vaccine groups. Graphs show GRA for individual rabbits (gray triangles) with the mean (black center line) and standard deviation (error bars) on different days for rabbits that were A) mock-vaccinated, B) APAB vaccinated with S4ΔaroD, C) APSB vaccinated with S4ΔaroD, D) SPAB vaccinated with S4ΔaroD. Mock-vaccinated includes historical controls, shown as open squares.
(TIF)

S6 Fig Changes in monocyte (MON) count after challenge in different vaccine groups. Graphs show MON for individual rabbits (gray triangles) with the mean (black center line) and standard deviation (error bars) on different days for rabbits that were A) mock-vaccinated, B) APAB vaccinated with S4DaroD, C) APSB vaccinated with S4DaroD, D) SPAB vaccinated with S4DaroD. Mock-vaccinated includes historical controls, shown as open squares.
(TIF)

S7 Fig Changes in platelet (PLT) count after challenge in different vaccine groups. Graphs show PLT for individual rabbits (gray triangles) with the mean (black center line) and standard deviation (error bars) on different days for rabbits that were A) mock-vaccinated, B) APAB vaccinated with S4DaroD, C) APSB vaccinated with S4DaroD, D) SPAB vaccinated with S4DaroD. Mock-vaccinated includes historical controls, shown as open squares.
(TIF)

S8 Fig Gross pathological changes after challenge. Figure shows gross pathology in lungs (A, C, E, G) and spleen (B, D, F, H) of rabbits at time of necropsy. Representative examples are shown. (A,B) mock vaccinated rabbit that succumbed to challenge; (C, D) APAB vaccinated rabbit that succumbed to challenge; (E, F) APAB vaccinated rabbit that survived challenge; (G, H) S4aroD scarification prime only vaccinated rabbit that survived challenge.
(JPG)

S9 Fig Fever and weight loss after aerosol prime/boost vaccination with S4ΔaroD or S4ΔguaBA. Rabbits were vaccinated with a prime and boost of S4ΔaroD or S4ΔguaBA by aerosol. Two groups of rabbits received S4ΔaroD in order to evaluate protection against higher challenge doses. Graphs show averaged daily group results (n = 6 per vaccine group and 4 mock-vaccinated controls); error bars indicate standard deviation. Vertical dotted lines indicate days for prime (day 0) and boost (day 14). A) Average fever response after prime (day 0) and boost (day 14) with S4ΔaroD and S4ΔguaBA compared to mock-vaccinated controls. The horizontal dotted line at 40°C indicates the threshold used for fever. B) Average weight change after vaccination with S4ΔaroD or S4ΔguaBA. A horizontal dotted line is shown at 0% weight change to make it easier to see where weight was lost in the vaccine groups.
(TIF)

S10 Fig Day 28 plasma IgG responses to prime only and prime-boost regimens. Figure shows day 28 post-vaccination plasma IgG response to rabbits that received S4DaroD via either scarification prime only (SP) or the different prime-boost regimens; APSB (aerosol prime, scarification boost), SPAB (scarification prime, aerosol boost), APAB (aerosol prime, aerosol boost) or mock-vaccinated rabbits. One-way ANOVA was performed to determine significance; * p < 0.05, *** p < 0.05, Δ 0.0001). Includes re-analyzed samples from Stinson et al (2016) and Reed et al (2014).
(TIF)

S11 Fig Day 28 plasma IgA responses. Figure shows day 28 plasma IgG titer (y axis) in relation to survival after challenge (x axis). One-way ANOVA found no correlation between IgA titer and survival after challenge (p = 0.4926, r2 = 0.2611).
(TIF)

S1 Table Relationship between dose and outcome. (DOCX)
