# Correction: Aerosol prime-boost vaccination provides strong protection in outbred rabbits against virulent type A *Francisella tularensis*

**DOI:** 10.1371/journal.pone.0207721

**Published:** 2018-11-15

**Authors:** The PLOS ONE Staff

The second author’s name is spelled incorrectly. The correct name is: Jennifer D. Bowling. The correct citation is: O’Malley KJ, Bowling JD, Stinson E, Cole KS, Mann BJ, Namjoshi P, et al. (2018) Aerosol prime-boost vaccination provides strong protection in outbred rabbits against virulent type A *Francisella tularensis*. PLoS ONE 13(10): e0205928. https://doi.org/10.1371/journal.pone.0205928

The last three authors, Karsten R. O. Hazlett, Eileen M. Barry, and Douglas S. Reed, should be noted as joint senior authors of this work. The publisher apologizes for the errors.
