# Supplementary figures and images for: Aerosol prime-boost vaccination provides strong protection in outbred rabbits against virulent type A Francisella tularensis
Source: PLoS One. 2018 Oct 22;13(10):e0205928. doi: 10.1371/journal.pone.0205928 (PMC6197691; doi:10.1371/journal.pone.0205928)

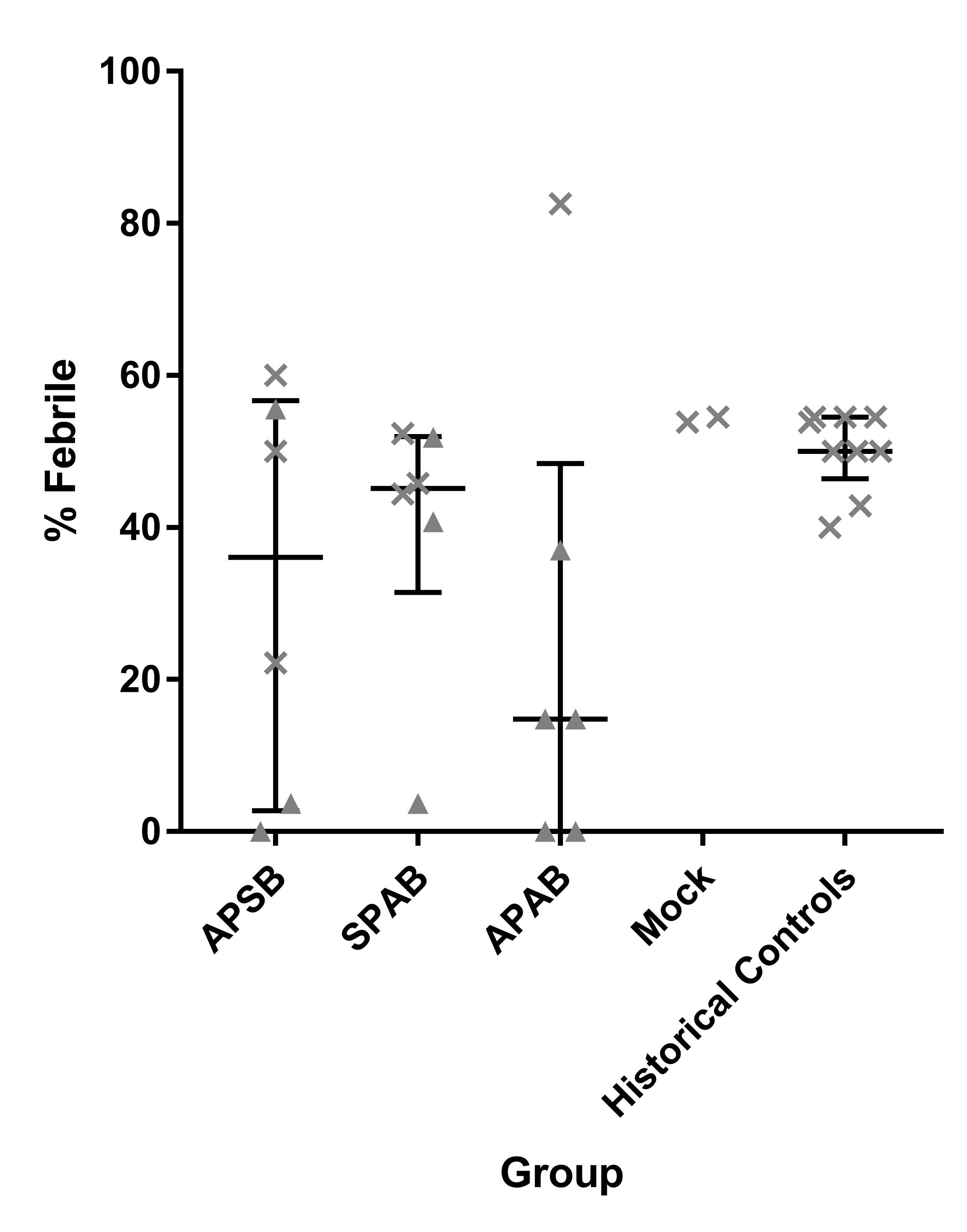

Supplement: S1 Fig — Graph shows the % of time points post-challenge that were scored as febrile (body temperature ≥ 40oC) for individual rabbits in the different vaccine groups. Individual rabbits that survived challenge are marked with gray triangles while rabbits that succumbed are marked with a gray X. Means and standard deviations are indicated by black bars for each group. (TIF) [file pone.0205928.s001.tif]

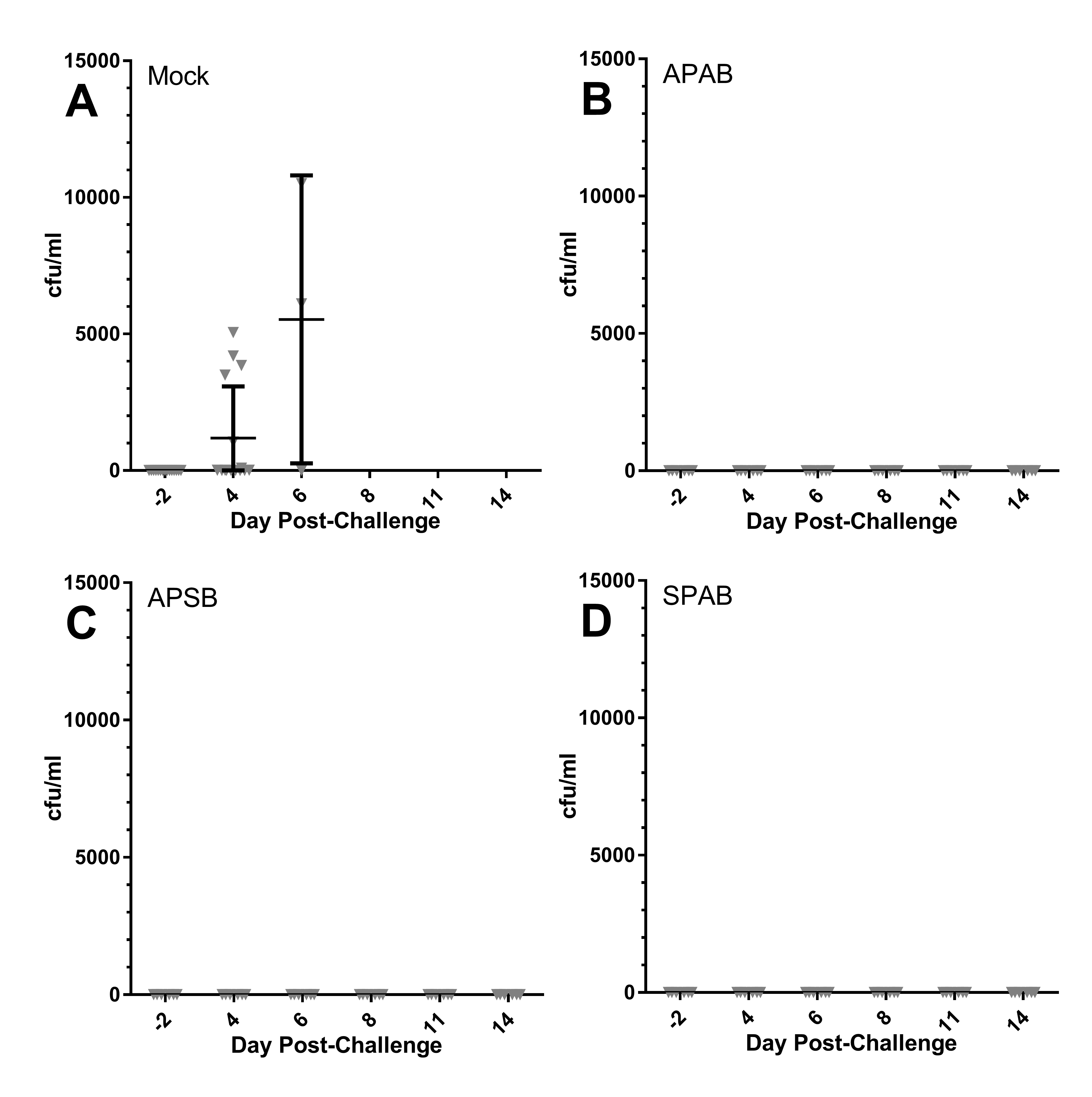

Supplement: S2 Fig — Graphs show bacteremia levels (in cfu/ml of blood) for individual rabbits (gray triangles) with the mean (black center line) and standard deviation (error bars) on different days for rabbits that were A) mock-vaccinated, B) APAB vaccinated with S4ΔaroD, C) APSB vaccinated with S4ΔaroD, D) SPAB vaccinated with S4ΔaroD. (TIF) [file pone.0205928.s002.tif]

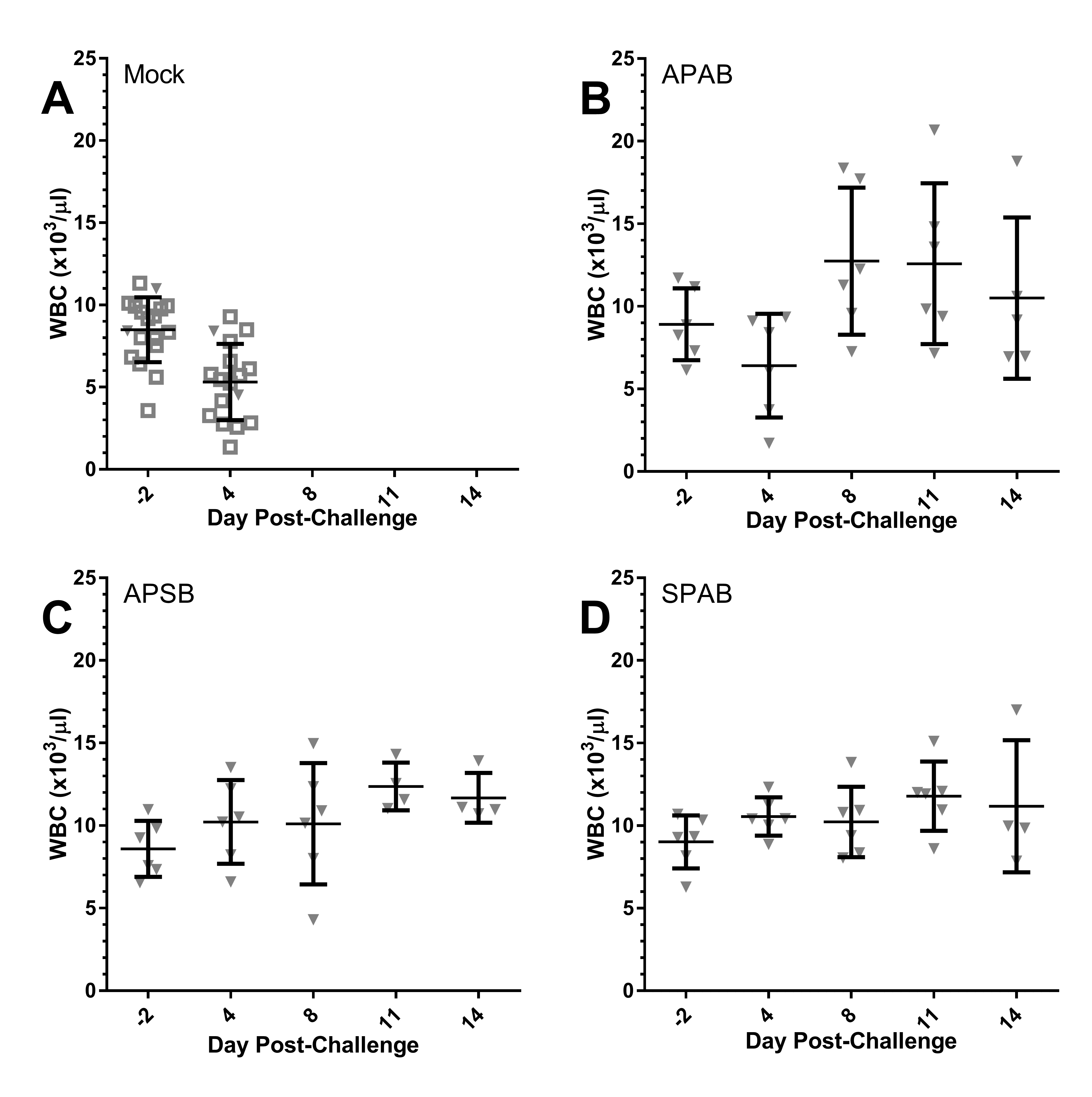

Supplement: S3 Fig — Graphs show WBC for individual rabbits (gray triangles) with the mean (black center line) and standard deviation (error bars) on different days for rabbits that were A) mock-vaccinated, B) APAB vaccinated with S4ΔaroD, C) APSB vaccinated with S4ΔaroD, D) SPAB vaccinated with S4ΔaroD. Mock-vaccinated includes historical controls, shown as open squares. (TIF) [file pone.0205928.s003.tif]

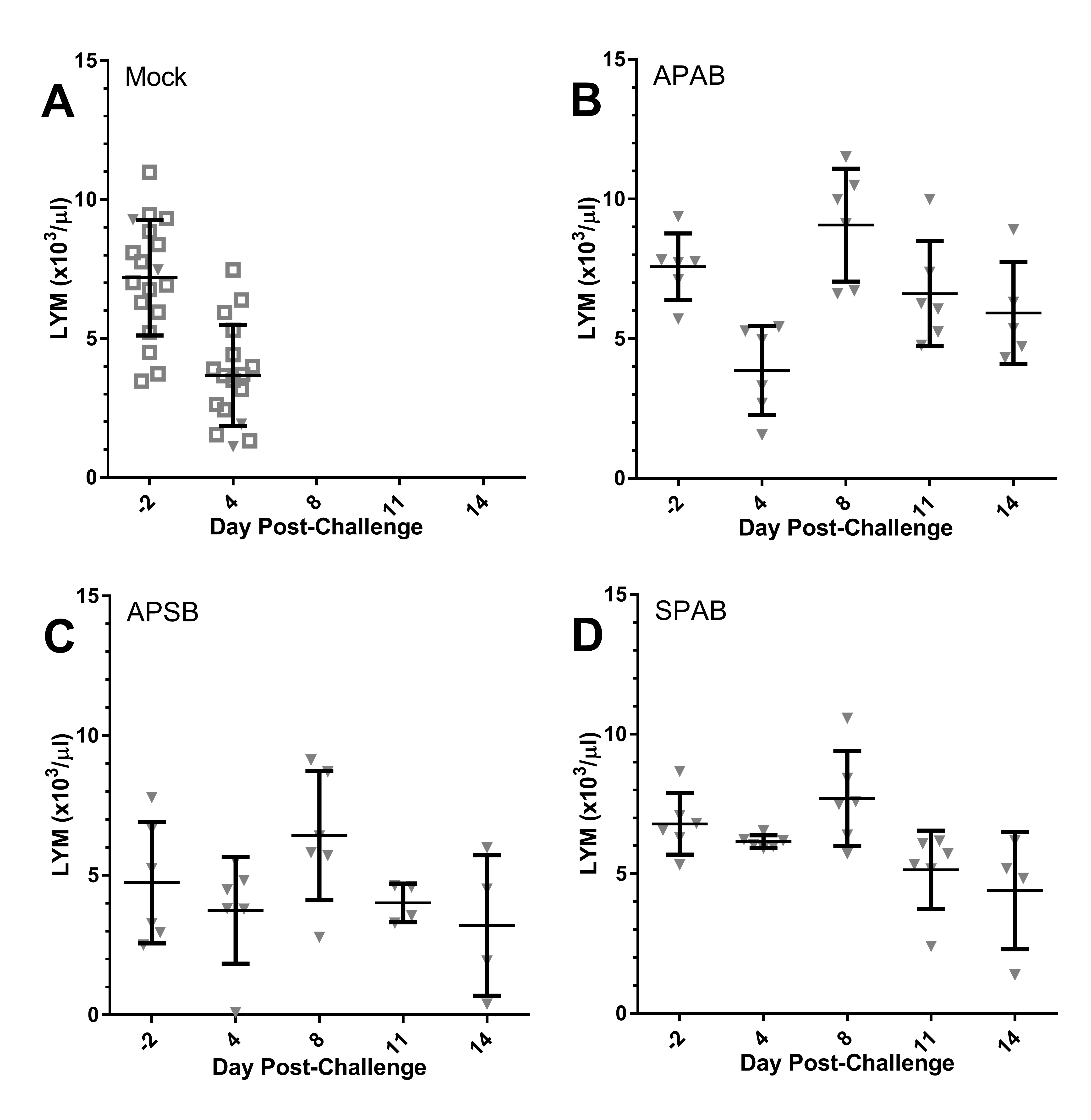

Supplement: S4 Fig — Graphs show LYM for individual rabbits (gray triangles) with the mean (black center line) and standard deviation (error bars) on different days for rabbits that were A) mock-vaccinated, B) APAB vaccinated with S4ΔaroD, C) APSB vaccinated with S4ΔaroD, D) SPAB vaccinated with S4ΔaroD. Mock-vaccinated includes historical controls, shown as open squares. (TIF) [file pone.0205928.s004.tif]

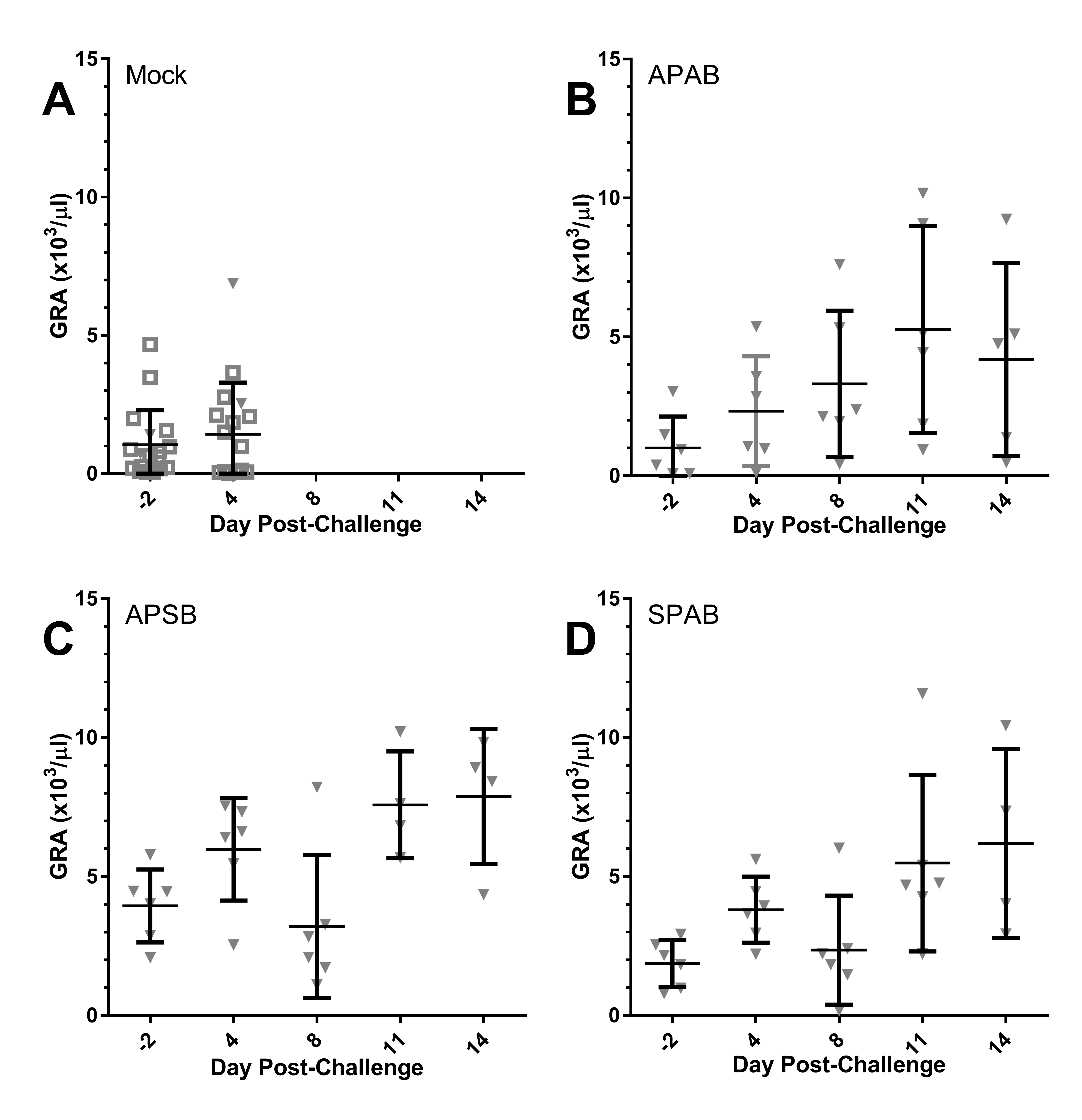

Supplement: S5 Fig — Graphs show GRA for individual rabbits (gray triangles) with the mean (black center line) and standard deviation (error bars) on different days for rabbits that were A) mock-vaccinated, B) APAB vaccinated with S4ΔaroD, C) APSB vaccinated with S4ΔaroD, D) SPAB vaccinated with S4ΔaroD. Mock-vaccinated includes historical controls, shown as open squares. (TIF) [file pone.0205928.s005.tif]

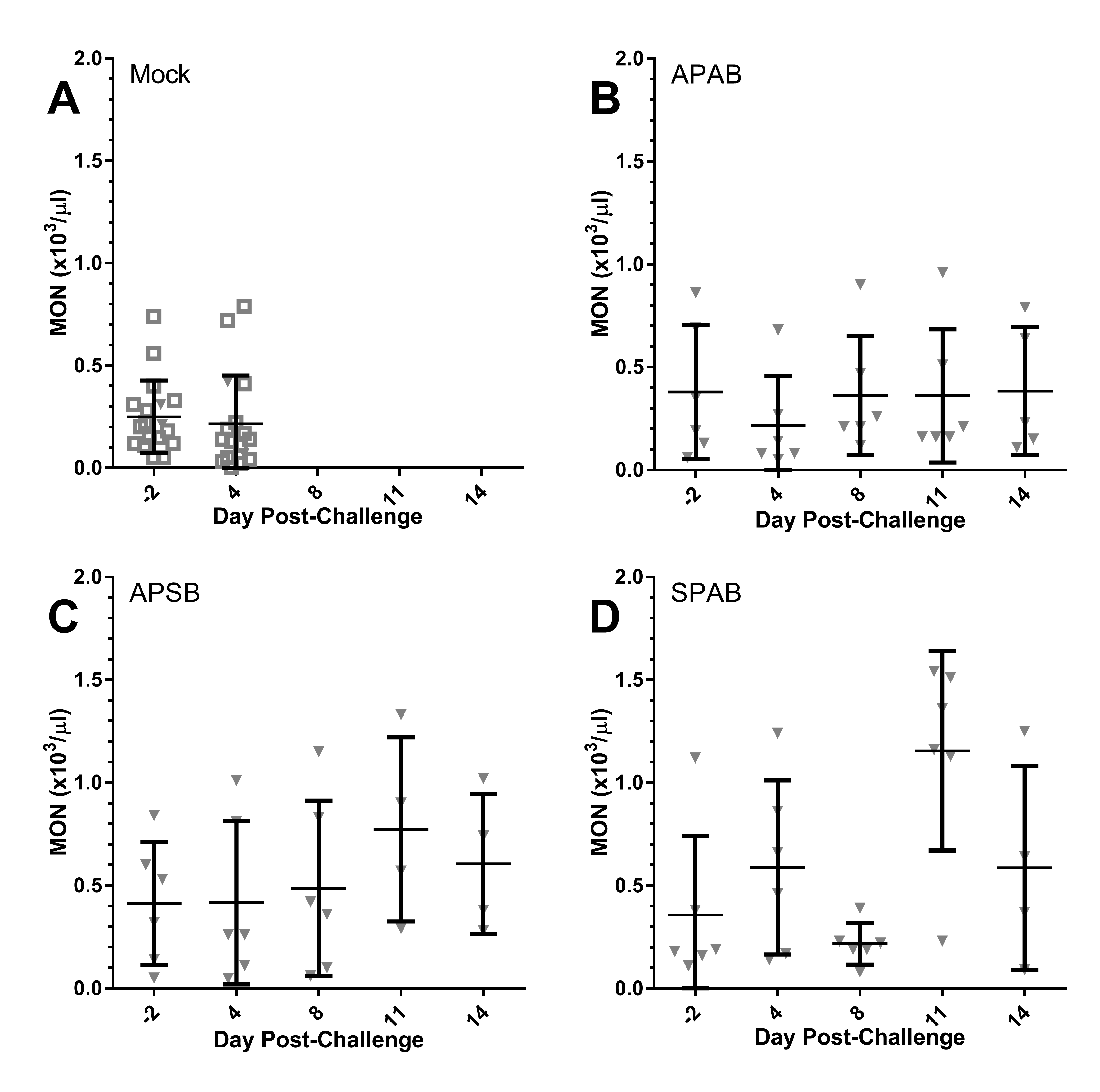

Supplement: S6 Fig — Graphs show MON for individual rabbits (gray triangles) with the mean (black center line) and standard deviation (error bars) on different days for rabbits that were A) mock-vaccinated, B) APAB vaccinated with S4DaroD, C) APSB vaccinated with S4DaroD, D) SPAB vaccinated with S4DaroD. Mock-vaccinated includes historical controls, shown as open squares. (TIF) [file pone.0205928.s006.tif]

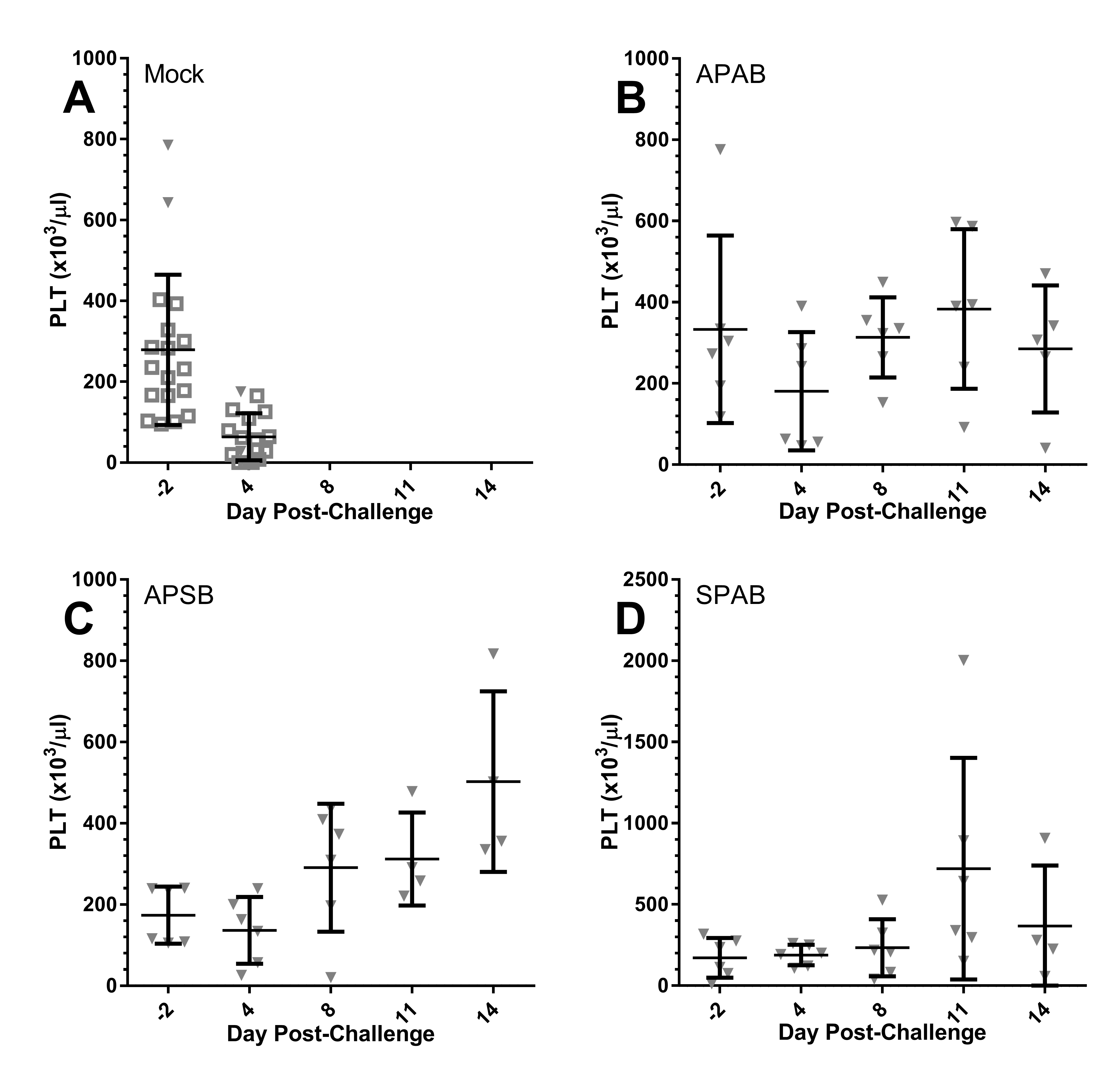

Supplement: S7 Fig — Graphs show PLT for individual rabbits (gray triangles) with the mean (black center line) and standard deviation (error bars) on different days for rabbits that were A) mock-vaccinated, B) APAB vaccinated with S4DaroD, C) APSB vaccinated with S4DaroD, D) SPAB vaccinated with S4DaroD. Mock-vaccinated includes historical controls, shown as open squares. (TIF) [file pone.0205928.s007.tif]

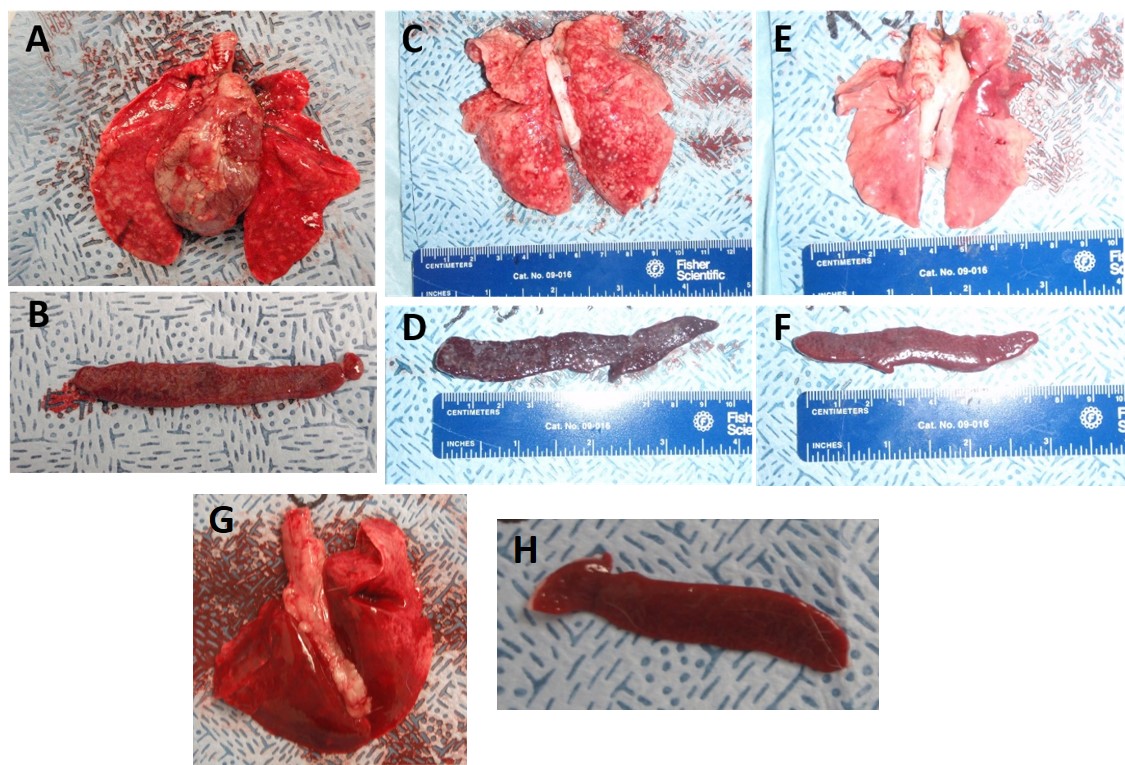

Supplement: S8 Fig — Figure shows gross pathology in lungs (A, C, E, G) and spleen (B, D, F, H) of rabbits at time of necropsy. Representative examples are shown. (A,B) mock vaccinated rabbit that succumbed to challenge; (C, D) APAB vaccinated rabbit that succumbed to challenge; (E, F) APAB vaccinated rabbit that survived challenge; (G, H) S4aroD scarification prime only vaccinated rabbit that survived challenge. (JPG) [file pone.0205928.s008.jpg]

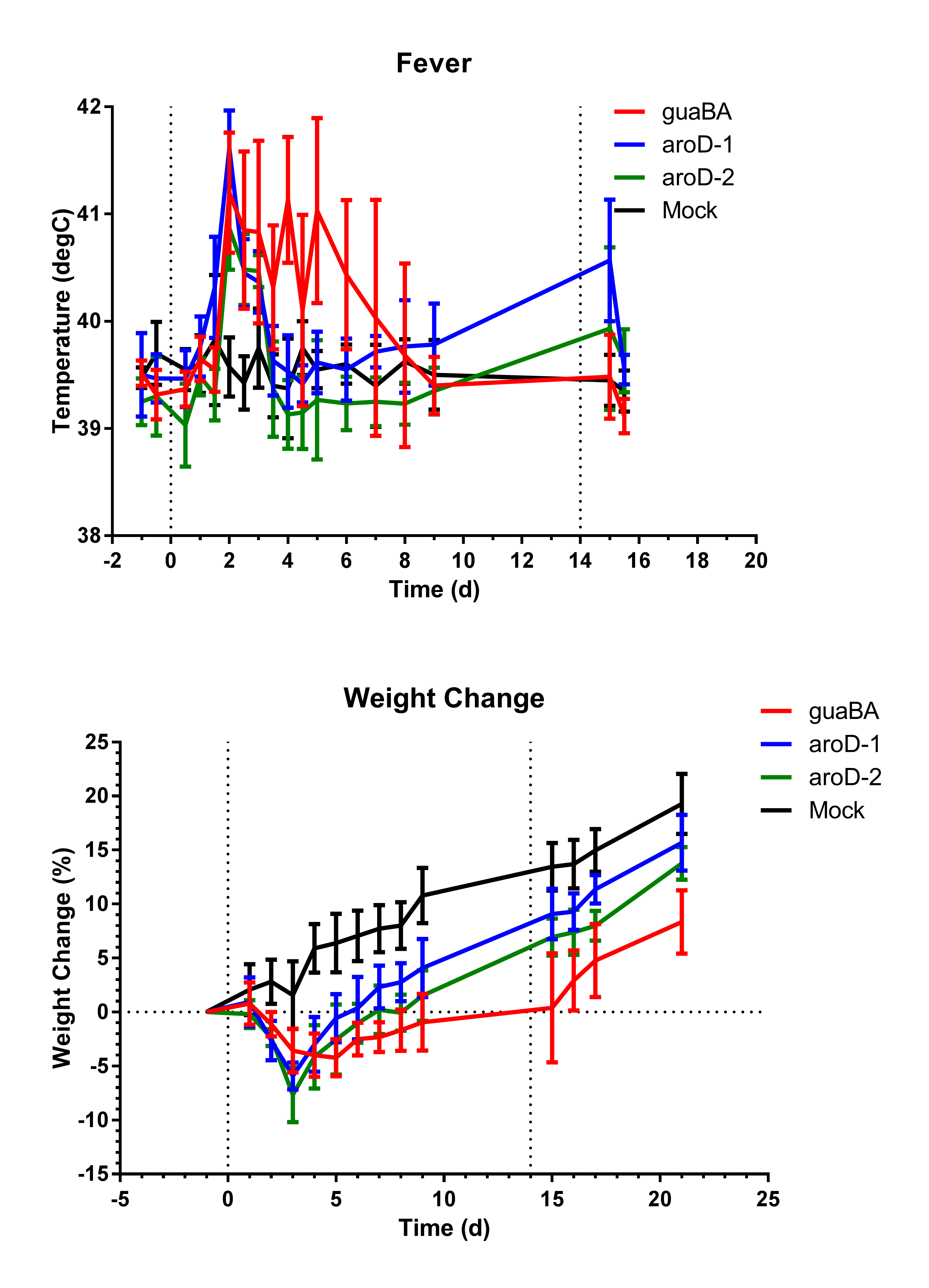

Supplement: S9 Fig — Rabbits were vaccinated with a prime and boost of S4ΔaroD or S4ΔguaBA by aerosol. Two groups of rabbits received S4ΔaroD in order to evaluate protection against higher challenge doses. Graphs show averaged daily group results (n = 6 per vaccine group and 4 mock-vaccinated controls); error bars indicate standard deviation. Vertical dotted lines indicate days for prime (day 0) and boost (day 14). A) Average fever response after prime (day 0) and boost (day 14) with S4ΔaroD and S4ΔguaBA compared to mock-vaccinated controls. The horizontal dotted line at 40°C indicates the threshold used for fever. B) Average weight change after vaccination with S4ΔaroD or S4ΔguaBA. A horizontal dotted line is shown at 0% weight change to make it easier to see where weight was lost in the vaccine groups. (TIF) [file pone.0205928.s009.tif]

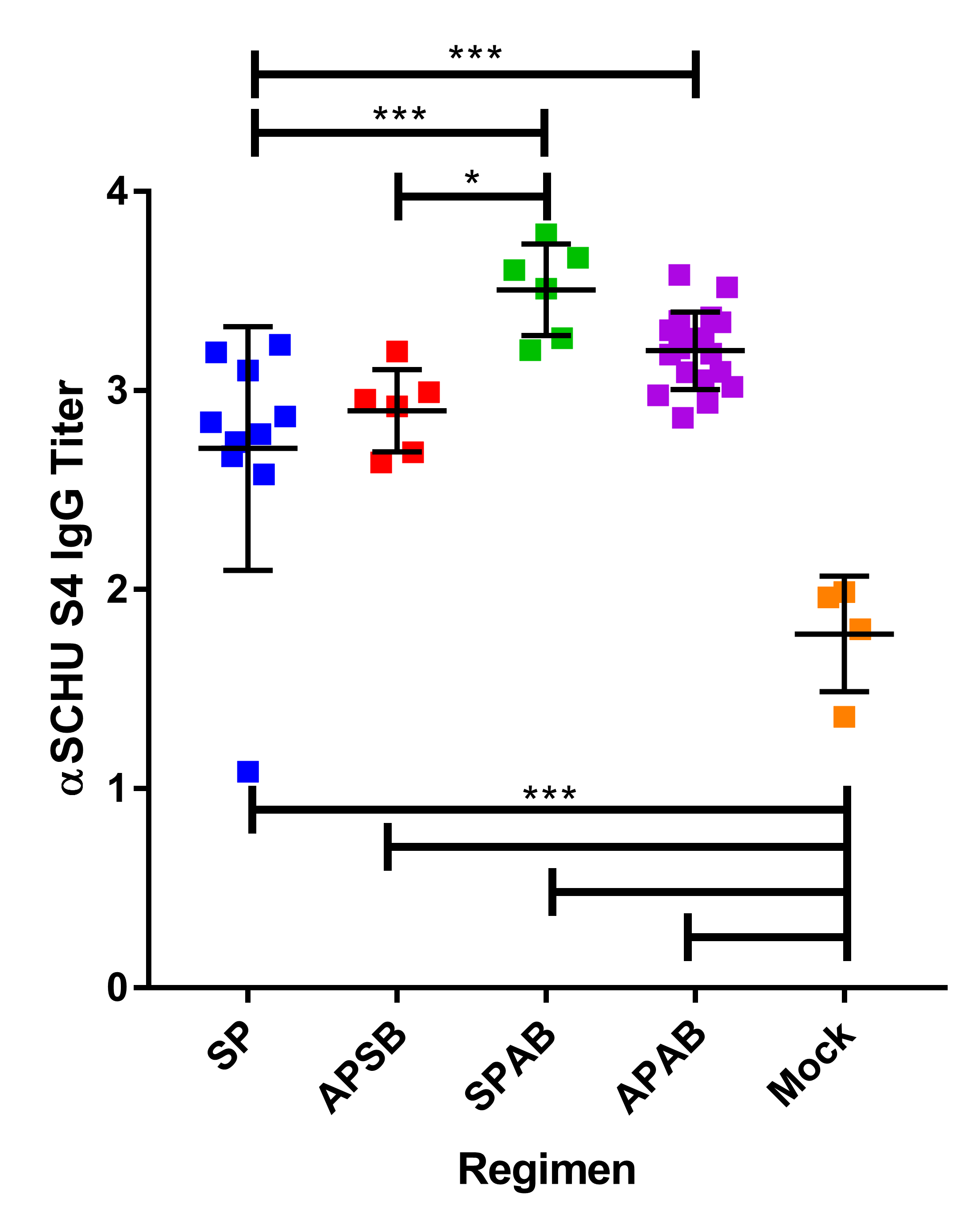

Supplement: S10 Fig — Figure shows day 28 post-vaccination plasma IgG response to rabbits that received S4DaroD via either scarification prime only (SP) or the different prime-boost regimens; APSB (aerosol prime, scarification boost), SPAB (scarification prime, aerosol boost), APAB (aerosol prime, aerosol boost) or mock-vaccinated rabbits. One-way ANOVA was performed to determine significance; * p < 0.05, *** p < 0.05, Δ 0.0001). Includes re-analyzed samples from Stinson et al (2016) and Reed et al (2014). (TIF) [file pone.0205928.s010.tif]

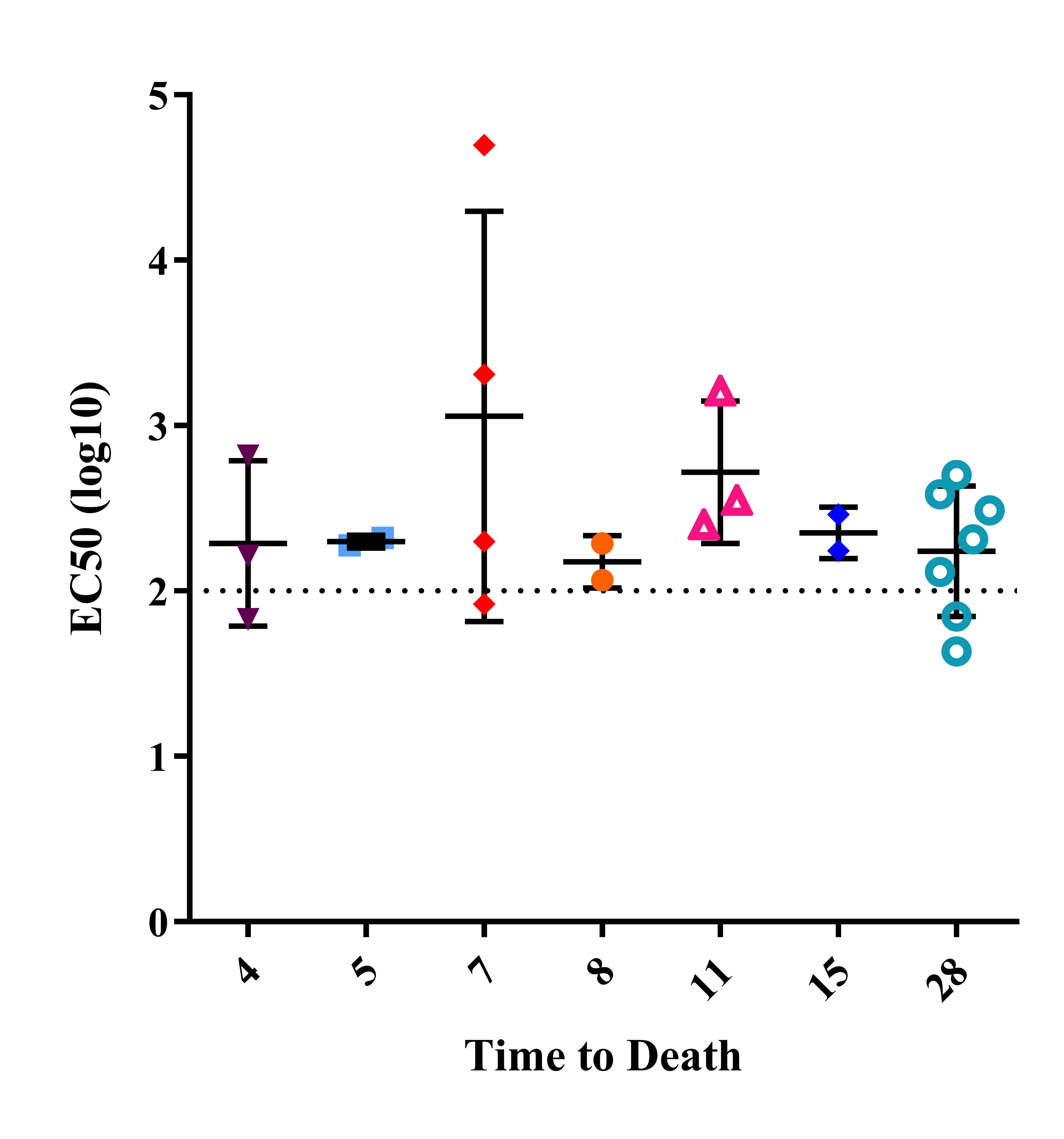

Supplement: S11 Fig — Figure shows day 28 plasma IgG titer (y axis) in relation to survival after challenge (x axis). One-way ANOVA found no correlation between IgA titer and survival after challenge (p = 0.4926, r2 = 0.2611). (TIF) [file pone.0205928.s011.tif]
