# Supplementary material for: Aerosol prime-boost vaccination provides strong protection in outbred rabbits against virulent type A Francisella tularensis
Source: PLoS One. 2018 Oct 22;13(10):e0205928. doi: 10.1371/journal.pone.0205928 (PMC6197691; doi:10.1371/journal.pone.0205928)
Supplement: S1 Table — (DOCX) [file pone.0205928.s012.docx]

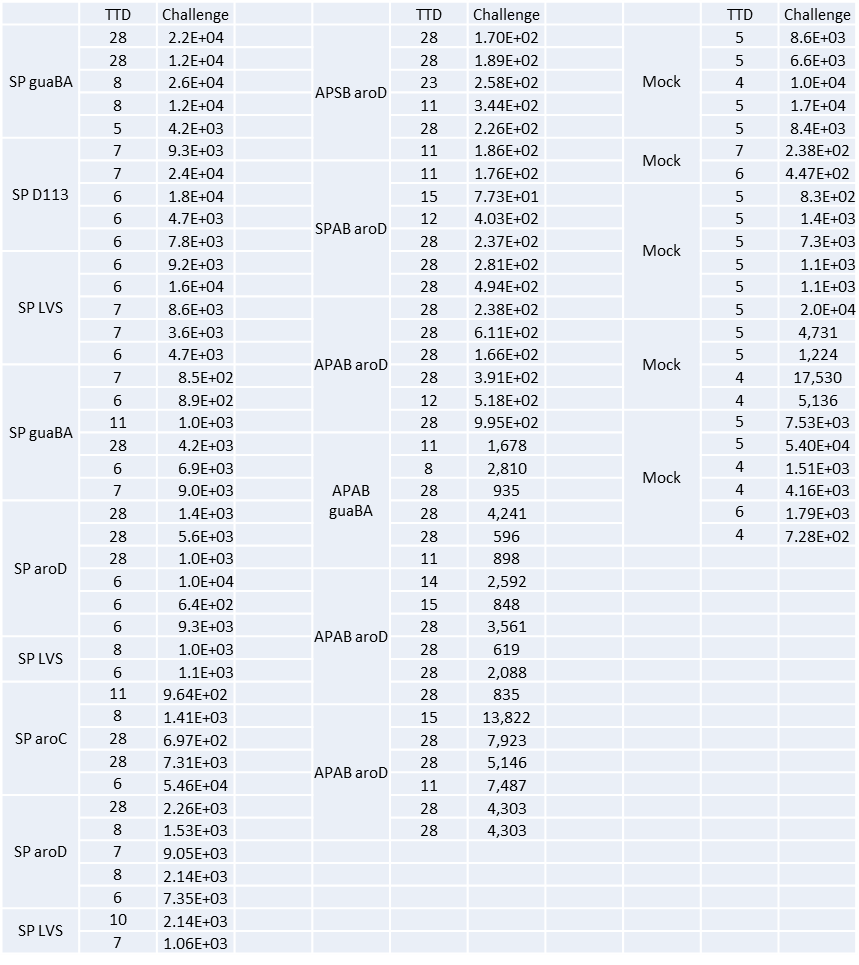
**Supplemental Table 1. Relationship between dose and outcome**

TTD = Time to Death

SCHU S4 challenge dose in cfu.

SP = scarification prime

SB = scarification boost

AP = aerosol prime

AB = aerosol boost
